# A Slug Flow Platform with Multiple Process Analytics Facilitates Flexible Reaction Optimization

**DOI:** 10.1002/advs.202308034

**Published:** 2024-01-25

**Authors:** Florian Wagner, Peter Sagmeister, Clemens E. Jusner, Thomas G. Tampone, Vidhyadhar Manee, Frederic G. Buono, Jason D. Williams, C. Oliver Kappe

**Affiliations:** ^1^ Center for Continuous Flow Synthesis and Processing (CC FLOW) Research Center Pharmaceutical Engineering GmbH (RCPE) Inffeldgasse 13 Graz 8010 Austria; ^2^ Institute of Chemistry University of Graz NAWI Graz, Heinrichstrasse 28 Graz 8010 Austria; ^3^ Boehringer Ingelheim Pharmaceuticals, Inc 900 Ridgebury Road Ridgefield CT 06877 USA

**Keywords:** Buchwald–Hartwig amination, data‐rich experimentation, flow chemistry, kinetics, self‐optimization

## Abstract

Flow processing offers many opportunities to optimize reactions in a rapid and automated manner, yet often requires relatively large quantities of input materials. To combat this, the use of a flexible slug flow reactor, equipped with two analytical instruments, for low‐volume optimization experiments are reported. A Buchwald–Hartwig amination toward the drug olanzapine, with 6 independent optimizable variables, is optimized using three different automated approaches: self‐optimization, design of experiments, and kinetic modeling. These approaches are complementary and provide differing information on the reaction: pareto optimal operating points, response surface models, and mechanistic models, respectively. The results are achieved using <10% of the material that would be required for standard flow operation. Finally, a chemometric model is built utilizing automated data handling and three subsequent validation experiments demonstrate good agreement between the slug flow reactor and a standard (larger scale) flow reactor.

## Graphical abstract

Using a flexible slug flow platform reaction optimization is explored using 1) self‐optimization, 2) design of experiments, and 3) kinetic profiling. This highly automated platform simultaneously allowed calibration/fitting of analytical models. A pharmaceutically‐relevant C‐N cross‐coupling reaction is optimized, with 6 independent variables, operated at high‐temperature/pressure conditions. Lower than 10% of starting material quantities are consumed compared to standard flow methods.

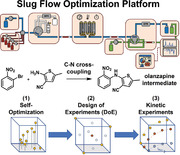

## 1 Introduction

**Figure 1:**
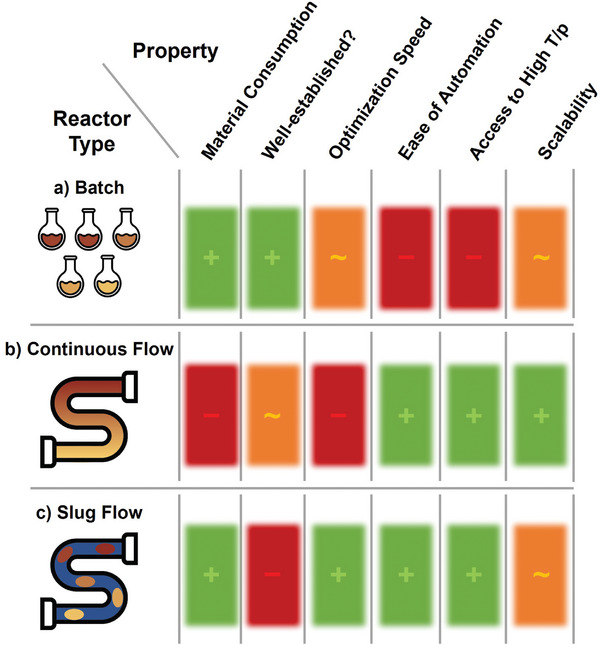
Comparison of reactor technologies for automated reaction optimization, highlighting their strengths, and weaknesses in different aspects. Green = favorable, orange = moderate, and red = unfavorable.

Modern synthetic chemistry labs are employing increasing levels of data generation and automation for reaction optimization in both batch^[^ ^1^ ^]^ and flow^[^ ^2^ ^]^ reactors. The resulting data‐rich environment generally leads to a larger number of experiments being conducted, allowing increased reaction understanding through modeling.^[^ ^3^ ^]^ This can be achieved in batch by using high throughput experimentation (HTE) techniques, with plates containing a large number of individual reaction vials or wells (**Figure** **1**a).^[^ ^4^ ^]^

Conducting flow reactions using standard approaches facilitates straightforward automation, but requires larger quantities of material for each reaction (Figure 1b). An operator will often wait for a standardized three times the reaction residence time to ensure that the system has reached a reliable steady state, even under “worst case” conditions. This is due to axial dispersion along the flow reactor, whereby the reaction segments of different compositions undergo some extent of mixing.^[^ ^5^ ^]^ In order to avoid this phenomenon, segments of material can be separated by an immiscible fluid (often gas), either forming a continuous segmented flow^[^ ^6^ ^]^, or individual reaction slugs. The latter approach has led to reactors that carry out individual flow experiments using significantly smaller quantities of reaction mixture, combining the benefits of both batch, and flow processing (Figure 1c).^[^ ^7^ ^]^ This has already been demonstrated for a small number of chemistries, such as photochemistry,^[^ ^8^ ^]^ homogeneous hydrogenation,^[^ ^9^ ^]^ and cross‐coupling.^[^ ^10^ ^]^

The availability of systems for carrying out automated reaction screening in flow has opened the door to a range of approaches for reaction optimization.^[^ ^11^ ^]^ Self‐optimization in flow^[^ ^12^ ^]^ generally employs Bayesian optimization algorithms to reach an optimum (or range of optima) in a “grey‐box” style, where no detailed reaction model can be extracted from the underlying Gaussian process models.^[^ ^13^ ^]^ Design of experiments (DoE) utilizes pre‐planned experiments to correlate reaction inputs to outputs, building a response surface model.^[^ ^14^ ^]^ Finally, kinetic modeling uses reaction time points to parameterize mechanistic reaction models, which describe the reaction rate(s) over time.^[^ ^15^ ^]^ Each approach has its merits and ideally a reactor should have the ability to carry out any combination, allowing the user to capitalize on their differing benefits.

Process analytical technology (PAT) has been used to dramatically accelerate reaction optimization in flow by providing results in real time.^[^ ^16^ ^]^ The majority of automated flow optimization examples rely solely on the use of chromatographic analysis methods: usually high‐performance liquid chromatography (HPLC).^[^ ^17^ ^]^ Although often carried out using a sample loop to inject directly from the reaction, the analysis times are relatively slow – in the range of 3–15 min depending on the chromatographic conditions employed. Despite its long analysis time, a high level of accuracy and low limit of quantification (LOQ) is achievable using HPLC.

Spectroscopic analysis, on the other hand, provides far more rapid results (<10 s per measurement), which can be highly advantageous for speeding up reaction cycles, especially when performing closed loop experiments. Despite the absence of any physical separation of compounds, overlapping signals can often be distinguished, and individually quantified using chemometric processing methods.^[^ ^18^ ^]^ By combining both a chromatographic and a spectroscopic PAT tool, the benefits of each can be accessed: spectroscopy for high temporal resolution of process stability and quantification, combined with chromatography for detailed reaction and impurity quantification.^[^ ^2^, ^19^ ^]^

The increased quantity of data generated can be problematic for standard workflows employed by chemists. Therefore, it is vital that automation is exploited wherever possible in such a reaction platform. Here, we report the use of a slug flow reactor platform, which utilizes only 300 µL of solution per reaction, with a high degree of automation, and two complementary PAT tools. The resulting system provides a high level of flexibility for a range of different optimization techniques, combined with automated data handling for rapid chemometric model building. We demonstrate the utility of this system for the optimization of a Buchwald–Hartwig coupling to form an active pharmaceutical ingredient (API) intermediate, using self‐optimization, DoE, and kinetic modeling.

## 2 Results and Discussion

### 2.1 Slug‐Flow Platform Design

The majority of slug flow reactor platforms make use of a liquid handler for reaction mixture makeup.^[^ ^7^, ^8^, ^9^, ^10^ ^]^ This takes specific quantities from multiple different vials with a syringe, then injects the mixture into a sample loop, allowing the mixture to be introduced to the reactor by switching a 6‐port valve. As an alternative approach, we simply used HPLC pumps to make up the reaction mixture of interest. Segments of gas as a separating medium were then injected using a sample loop. The use of pumps has the advantage of requiring less complex equipment and operating algorithm, as well as fewer individual steps, allowing all components of the reaction mixture to be mixed instantaneously and transferred to the reactor more rapidly. In fact, using this approach, the reaction slug is made up and gas segments are injected in under 1 min. On the other hand, changing reaction components would need to be done manually, with a cleaning step in between. This present platform is best suited to later stage reaction development, where the categorical variables (e.g., solvent and catalyst identity) have already been selected. The quantity of inert gas can be easily varied by changing the sample loop size as well as substituted for an inert liquid phase (e.g., perfluorinated liquids).

**Figure 2:**
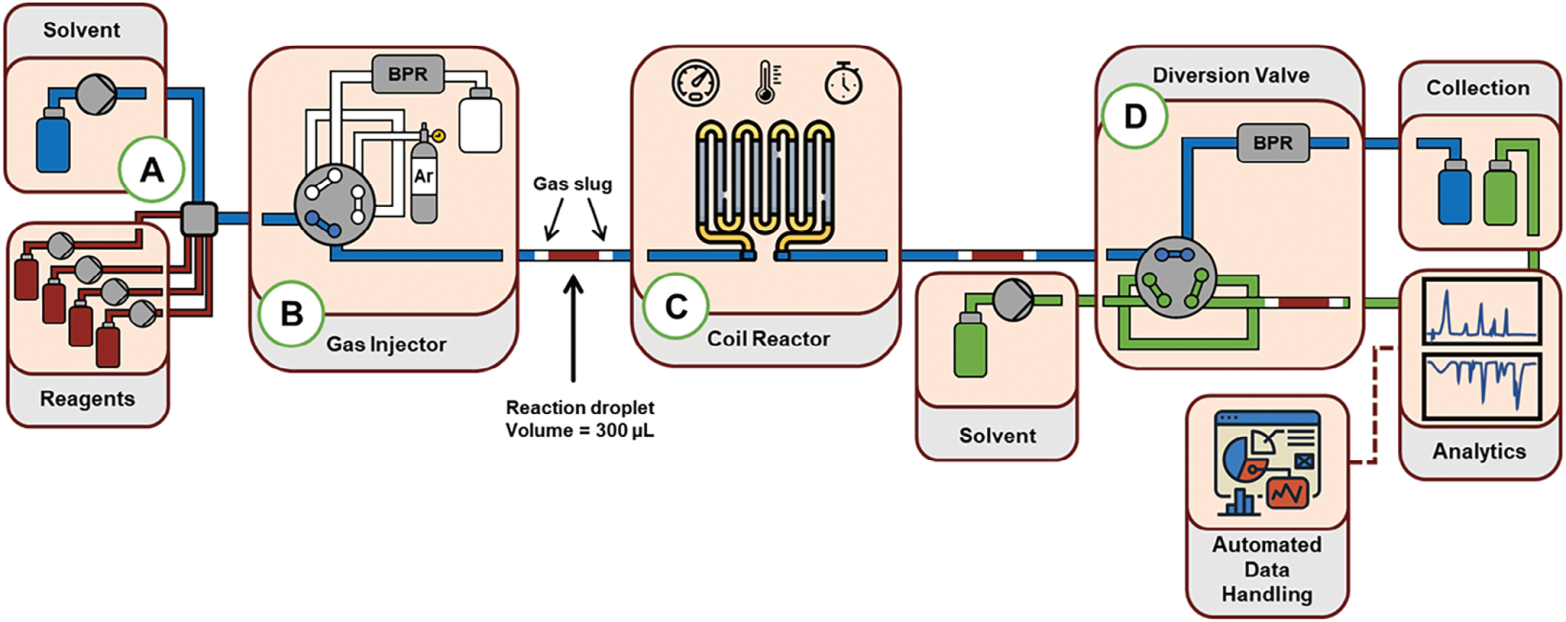
Schematic representation of the developed reactor platform. a) Reagent and solvent pumps, combining in a 6‐way mixer. b) Sample loop for gas injection. c) Heated coil reactor. d) 6‐port valve to direct the reaction slug for analysis.

As an overview of the system, five HPLC pumps were used to deliver 4 reagent mixtures and 1 solvent stream into a mixing unit (**Figure** **2**a). The default position delivered solvent only. When beginning a reaction, the pumps were switched on at the necessary flow rates. Once steady pump flow rates were achieved, a slug of inert gas (45 µL volume) was injected, by switching an automated 6‐port valve (Figure 2b). The reaction slug was then pumped to make up a volume of 300 µL, followed by injecting a second slug of gas. The reaction slug was of constant concentration across its length and the slugs of gas prevented any axial dispersion/dilution by the carrier solvent. A brief study was performed to examine different reaction slug sizes, mixing conditions and reaction residence times (see Figure S22, Supporting Information), in which 300 µL was found to provide reproducible and homogeneous reaction slugs.

When optimizing a reaction with multiple variables in a wide design space, the required flow rates are often impossible to achieve, due to high and low flow rate combinations not being accessible by the pumps. However, in this approach, the total flow rate for reaction slug formation is constant, since the residence time is determined by the solvent flow rate after slug formation. Importantly, this allowed the residence time to be decoupled from the flow rates used to make up the reaction slug. Numerous tests were carried out to ensure proper operation of the slug flow reactor system, focusing on the accurate delivery of each reagent to reach the desired reaction slug composition (see Supporting Information for details).

The reaction slug was then pushed through the heated reactor coil by a stream of solvent (Figure 2c), whose flow rate was adjusted to achieve the desired residence time. A solvent carrier was chosen in place of gas due to the far smaller change in volume with temperature changes. This simplifies the residence time calculation and ensures that it is accurately achieved. Furthermore, the flow of solvent served to continually flush/clean the reactor, meaning that a separate cleaning step was not necessary.

After the heated reactor zone, the reaction slug (with gas slugs) was flowed into a sample loop via a second automated 6‐port valve (Figure 2d). Once inside the loop, the valve was switched to deliver the reaction slug into the analytics pathway. Within this analytics pathway, a constant flow rate was used to push the slug, again decoupling this from the residence time in the reactor itself, and meaning that flow rate‐sensitive analytical techniques (such as inline NMR) would be usable without issue.^[^ ^20^ ^]^ This separation also provided the advantage that the next reaction iteration may in theory be initiated even before the analysis step, essentially allowing the two to run in parallel, and affording significant time savings. The analytics pathway could also be paused, allowing additional analysis (e.g., repeat HPLC injection) to be made.

A key feature of this reactor setup was the synergistic use of two PAT tools: one spectroscopic and one chromatographic. In this specific configuration, the analytics pathway first flowed through a Fourier transform infrared (FTIR) spectrometer (Mettler Toledo, ReactIR 15) flow cell, which recorded spectra every 5 s. When the reaction slug was detected by this instrument, the control software sent a signal (after a defined time) to the ultra‐high‐performance liquid chromatography (UHPLC) to inject and begin measuring. By triggering the injection in this manner, accurate sample injection could be ensured reproducibly, with no influence of reaction parameters, and timing. The resulting data was also used in a complementary manner for chemometric modeling (as demonstrated later in this manuscript).

### 2.2 Optimization Approach

**Figure 3:**
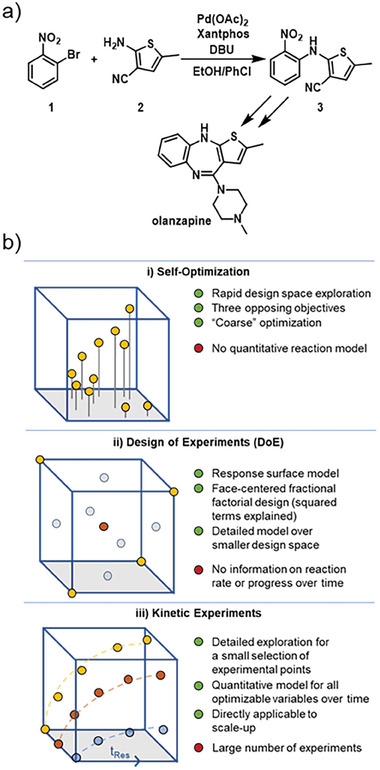
a) Buchwald–Hartwig amination of **1** by **2**, forming olanzapine intermediate **3**. b) Schematic overview of the optimization approaches demonstrated here: i) self‐optimization; ii) DoE; and iii) kinetic experiments.

In order to demonstrate the utility of the developed platform for reaction optimization, three different experiment types were performed. The reaction to be optimized was chosen to ensure a high degree of mechanistic complexity and a large number of optimizable variables. The Buchwald–Hartwig amination of aryl bromide **1** with thiophene‐based aniline **2** produces diaryl amine **3** – an intermediate in the synthesis of antipsychotic medication, olanzapine (**Figure** **3**a). Amination of the more reactive aryl iodide has been reported in batch and flow,^[^ ^21^ ^]^ however, use of the cheaper aryl bromide substrate was not yet known. Buchwald–Hartwig couplings represent an ideal reaction to optimize for an individual target substrate, due to their vastly varying performance with different substrates.^[^ ^4c^ ^]^ In this case, 1,8‐diazabicyclo(5.4.0)undec‐7‐ene (DBU) was employed as a strong organic base to ensure a homogeneous reaction mixture, as has been previously reported for such coupling reactions.^[^ ^22^ ^]^

The chemistry in question was performed with up to six independent optimizable variables: 1) loading of amine **2**; 2) reaction concentration (based on **1** as the limiting reagent); 3) residence time; 4) reaction temperature; 5) loading of base (DBU); and 6) loading of catalyst. This resulted in a challenging optimization problem with a high degree of complexity. For practical reasons, the ratio of ligand (Xantphos) to catalyst was kept constant at 1.5, but could also be envisioned as a seventh optimization variable.

The optimization approach demonstrated here consisted of three experiment types (Figure 3b). First, a multi‐objective self‐optimization campaign was carried out using the Thompson sampling efficient multi‐objective (TSEMO) Bayesian optimization algorithm.^[^ ^23^ ^]^ Our approach used this experiment type to map the broadest possible design space (“coarse optimization”), where the region of interest was found. Based on this data, it was also possible to find the general influence of the optimizable variables, and allowing two to be fixed at a constant value for the next optimization stage, DoE.

The knowledge gained in the self‐optimization experiments facilitated a more focused DoE study, by reducing the number of optimizable variables to decrease experimental burden, as well as narrowing the variable ranges to improve model accuracy in this narrower range. Therefore, it was feasible to perform a full factorial design (2^n^ = 16 experiments), with additional “face points” (2n = 8 experiments), defining the non‐linear effect of optimizable variables. It was envisaged that this set of experiments would provide a response surface model, quantifying the effect of each examined variable.

Finally, based on the results from the first two stages, a kinetic study was carried out in the slug flow platform. Six different reaction conditions were each reproduced at six residence times. The resulting time course data was used to parameterize a kinetic model. In order to validate the optimization approaches, a larger scale reactor was constructed and a setpoint was selected from each of the three optimization approaches, to ensure that the results could be reproduced in a standard flow reactor configuration.

### 2.3 Self‐Optimization

Self‐optimization was performed using the TSEMO algorithm,^[^ ^23^ ^]^ with six optimizable variables (whose ranges were set to be as broad as possible) and three objectives: 1) yield (%); 2) space‐time yield (kg/(L*h)); and 3) cost of reaction, based on the solutions consumed (€). These three objectives were chosen with the aim of optimizing in different directions. This multi‐objective optimization approach can serve to map out the complex relationships between different performance indicators and provides the optimization algorithm with an incentive to explore more operationally‐favorable reaction conditions. Complementary to a classical yield optimization, space‐time yield provides a productivity measure, which favors faster reactions. The cost objective aims to reduce the excess of reagents used, thus waste generated. Therefore, this acts as both an economical and sustainability objective.

To initialize the self‐optimization, a Latin hypercube (LHC) was used, performing twelve (2n = 12) experimental iterations, evenly spread throughout the design space. Thereafter, a further 48 iterations, guided by Bayesian optimization, were performed, providing a total of 60 results. Despite operating at relatively high reaction concentrations, the self‐optimization campaign required only a minimal quantity of starting materials (e.g., 3 g aryl bromide **1**, 0.12 g Pd(OAc)_2_), calculated to be 7% of the theoretical quantity required for comparable steady state experiments. The time taken for each reaction (including slug makeup and UHPLC analysis) was roughly the residence time + 6.5 min. The 60 self‐optimization experiments were carried out in ≈12 h, which represents an effective use of time, especially considering the fully automated nature of the platform.

**Figure 4:**
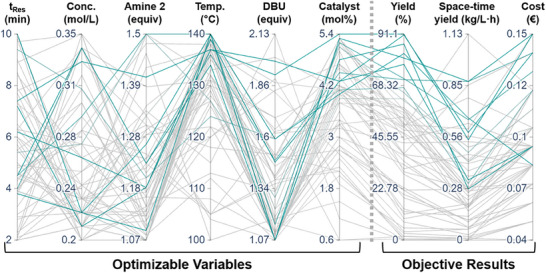
Results of self‐optimization experiments displayed as a parallel coordinate plot. Each line represents an experiment, relating the optimizable variable setpoints to the corresponding outputs. For ease of visualization, only experiments resulting in >65% yield are highlighted in green.

The parallel coordinate plot (**Figure** **4**) facilitates visualization of data with high dimensionality, which is difficult to comprehend in 2D and 3D graph types. The experiments providing >65% yield (9 results) are highlighted in green. Within this sub‐selection of results, it can clearly be seen which factors have a significant influence. It is not necessary to use a long residence time, as evidenced by the experiment achieving good yield at *t*_Res_ <4 min. Similarly, it can be seen that there is no real trend when varying reaction concentration, amine **2** loading and DBU loading. Conversely, almost all of the high yielding experiments required a high‐temperature and catalyst loading.

The relationships between the three objectives can also be visualized using this technique: the high yielding experiments can also achieve a high space‐time yield, but often result in a high cost. The full parallel coordinate plot can be found in the Supporting Information. Within the experiment set, a maximum yield of 91% was achieved or, under different conditions, a maximum space‐time yield of 1.13 kg/(L*h). In addition, an experimental point was repeated several times during the self‐optimization campaign, in order to ensure reproducibility and alert the operator to any changes in results over time (e.g., decomposition of feed solutions).

### 2.4 Design of Experiments

DoE is widely utilized in industrial process development labs, in order to rapidly gain understanding, optimize, and build response surfaces for reactions.^[^ ^13b^ ^]^ A full factorial design requires 2^n^ experiments, which is experimentally demanding in cases with a large number of optimizable variables, such as this. By holding some variables constant, the number of experiments can be significantly decreased. Addition of so‐called face points (face‐centered design) allows the non‐linear terms to be specifically described, which is not possible using a standard factorial design.

Following on from the self‐optimization experiments, the number of optimizable variables was reduced. Residence time was fixed to a constant value (*t*_Res_ = 6.5 min), since full conversion was already observed in self‐optimization experiments at this time and keeping a constant residence time allows better comparison between results in a DoE campaign. To work under the most desirable conditions, concentration was also set to a constant level (concentration = 0.33 mol L^−1^), maximizing space‐time yield, and minimizing solvent waste.^[^ ^24^ ^]^ This allowed the remaining 4 variables to be examined in greater detail, without an excessive number of experiments. A face‐centered full factorial design (2^n^ “corner points” + 2n “face points”) was chosen, for a total of 24 experiments, as well as center points to test reproducibility.

The parameter space was also narrowed down, to ensure that the more productive part of the design space was in focus. Modeling a larger area is less likely to provide a good response surface model, due to the differing behavior in different areas of the design space. Only high‐temperatures (130–140 °C) were examined, as well as high catalyst loading (4.3–5.4 mol.%). Amine **2** and DBU loading were kept relatively flexible (1.07–1.28 equiv and 1.07–1.61 equiv, respectively). These chosen parameter ranges should lead to high yielding reactions, whilst keeping reagent wastage low.

**Figure 5:**
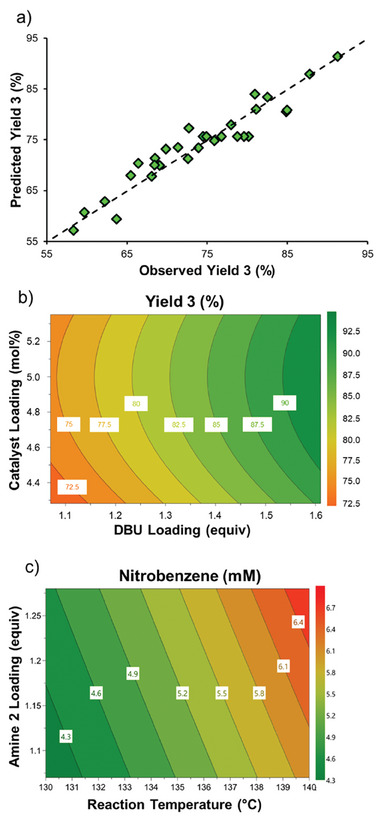
Selected results of DoE study. a) Predicted versus observed results for the reaction yield, showing excellent agreement (R^2^ = 0.904). b) Surface plot showing the predicted yield versus catalyst loading and DBU loading (amine **2** loading = 1.28 equiv, temperature = 140 °C). c) Surface plot showing the predicted quantity of nitrobenzene formed versus amine **2** loading and temperature (DBU loading = 1.61 equiv, catalyst loading = 4.28 mol.%).

The resulting model for reaction yield provided an excellent fit (**Figure** **5**a, R2 = 0.904, Q^2^ = 0.829), as well as good reproducibility (six center points provided between 74 and 80% yield, despite being run across two different days with different stock solutions). DBU loading was found to have a significant positive effect on yield, within the examined range (Figure 5b). This discrepancy with the previous self‐optimization results could be due to the narrower range examined here (1.07–1.61 vs 1.07–2.13 equiv). Furthermore, the catalyst loading had a non‐linear effect, resulting in optimal yield at a moderate loading (≈5 mol.%).

Response surface models were built for two observed low‐level impurities, one of which arising from protodehalogenation (nitrobenzene) and the other of unknown structure. Although nitrobenzene formation was only observed at low level in all cases (<7 mm concentration), its formation was clearly seen to be promoted by higher temperature and amine **2** loading, whilst disfavored by high catalyst loading (Figure 5c). Meanwhile, the unknown impurity was mostly formed due to a high DBU loading. Further details for all DoE results and models can be found in the Supporting Information.

### 2.5 Kinetic Data Acquisition

**Figure 6:**
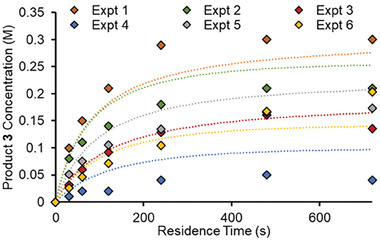
Plot showing product **3** concentration in six different time‐course experiments. Points denote experimental measurements, whilst dotted lines denote predicted values using the fitted kinetic model.

As a final optimization approach, a selection of six experimental conditions were selected, and based on the data collected thus far (see Supporting Information for details). These conditions were selected to examine variation in all of the optimizable variables. For each of these conditions, six experiments were carried out, each at a different residence time, between 0.5 to 12 min (**Figure** **6**, points). The resulting data was then used to parameterize a kinetic model, which providing rate data, which had not been considered thus far. The kinetic model consisted of a catalytic cycle, with four productive reactions to provide the desired product. Initial fitting suggested only the oxidative addition step to be rate‐limiting (fitted with an activation energy and pre‐exponential factor), therefore the subsequent steps were fixed with an arbitrary high bimolecular rate constant. To account for catalyst deactivation and impurity formation, one side reaction pathway was added and fitted with a temperature‐independent rate constant.

These three parameters were fitted to all experimental data in parallel, resulting in a good fit (Figure 6, dotted lines). This kinetic model was then successfully used to predict the product concentrations for each of the 60 self‐optimization experimental results. Despite the model being fitted using a smaller subset of the design space, the root‐mean‐square error (RMSE) for these predictions was 34.9 mm. For further validation, the DoE results were also predicted, resulting in a lower RMSE of 25.0 mm. This reinforces the expectation that the kinetic model provides more accurate predictions within the section of the design space of interest. See the Supporting Information for the catalytic cycle and further discussion of kinetic fitting and predictions. Using such a kinetic model can accelerate further reaction optimization, by facilitating in silico experimentation and optimization.

### 2.6 Chemometric Modeling and Validation Experiments

**Figure 7:**
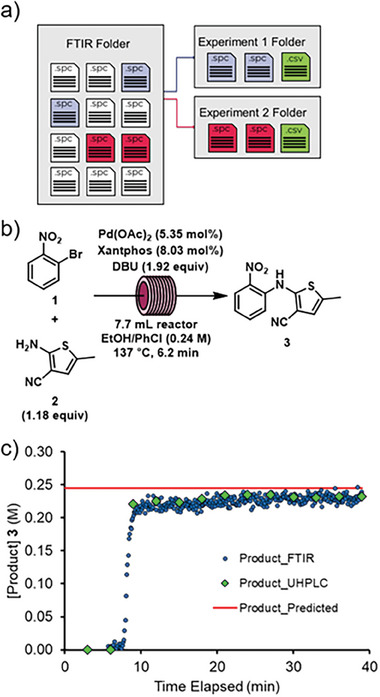
a) Schematic depiction of the algorithm workflow used to sort incoming data and assign the corresponding concentration values for each experiment. b) Reaction conditions chosen for validation of self‐optimization experiments in continuous flow. c) Plot showing concentration of product **3** measured by both UHPLC and FTIR, during validation of kinetic model.

The combination of two PAT tools can, with automated data management, be used to minimize the burden of chemometric model generation. For the most accurate partial least squares (PLS) regression model, it is preferable for genuine reaction samples to be used for calibration. A python script was developed to automatically sort FTIR spectra for each experiment and assign the UHPLC recorded concentrations of starting materials and products in a csv file (**Figure** **7**a). The dataset generated during the self‐optimization experiment was used to rapidly construct a PLS model with minimal manual data handling steps. The models for aryl bromide **1**, amine **2** and product **3** had low RMSE (cross validation) of 23, 45, and 14 mm, respectively. This model would now allow inline quantification of the three major reaction species in real time, without the need for chromatography.

To validate the data obtained from the slug flow reactor and the optimization approaches used here, three experiments were carried out in a larger flow reactor (3.6‐fold increase in volume) with continuous delivery of the reaction mixture (Figure 7b). The experiments consisted of: 1) a repeat of a self‐optimization iteration; 2) predicted high‐yielding conditions from the DoE; and 3) predicted high‐yielding experiment at short residence time from the kinetic model.

The first validation experiment provided a stable value of 77.4% yield, compared with the previously recorded 91.1%. This discrepancy may be due to the slight changes in heat‐ and mass transfer between the two setups. The larger diameter tubing used in this setup (1/8″ vs 1/16″ outer diameter) may result in a shorter reaction time at the desired temperature due to poorer heat transfer in the heated reactor section. This larger diameter, and absence of gas slugs, may also provide poorer radial mixing.

The second experiment served to also validate the DoE response surface model. An optimal set of conditions, based on the response surface, was predicted to provide 90.2% yield. The resulting 83.5% yield showed a discrepancy of 6.7% – lower than that observed in validation experiment 1. The final validation experiment was against the kinetic model (Figure 7c), whereby 74.2% yield was predicted with a short residence time of only 4.2 min. The corresponding experimental result was 70.1%, representing a discrepancy of only 3.1% (lower than those observed in the previous two experiments). Full details of validation experiments are available in the Supporting Information.

## 3 Conclusion

We have demonstrated a workflow using an automated slug flow reactor platform for the optimization of a cross‐coupling reaction with high dimensionality. The platform was designed for maximum flexibility, whilst incorporating both spectroscopic and chromatographic PAT instruments. Its accuracy and reproducibility were examined using different flow rates and slug sizes, before beginning experimental optimization. This flow setup offered distinct advantages compared to batch high throughput experimentation, since the iterative nature of examining one reaction condition at a time allows for more responsive modeling – particularly in self‐optimization campaigns. Furthermore, the benefits of flow chemistry in high‐temperature/pressure processing could be harnessed to dramatically decrease reaction times.

The optimization was performed using a combination of three commonly‐employed methods: self‐optimization, DoE, and kinetic modeling. Within 60 experiments, self‐optimization provided a range of experimental conditions to fulfill a balance between yield, productivity, and cost. The DoE experiments provided a useful response surface model, with a reduced number of variables, for both yield and impurity content. An excellent kinetic model was then built, based on 36 experimental points and validated against the self‐optimization and DoE data. Finally, the FTIR data collected during the self‐optimization experiments was utilized to rapidly build a PLS model to quantify the three main reaction species. Three validation experiments were then performed in a larger (“standard”) flow reactor to validate the reaction conditions, model predictions, and PLS model. The reaction optimization discussed in this manuscript represents a complex and pharmaceutically‐relevant example and it is envisaged that the platform may have a broad range of applications in pharmaceutical process development.

The three demonstrated optimization approaches each serve a different purpose, but altogether contribute to a complete overall picture of the reaction. Self‐optimization was used to gain an overview of the reaction performance and find the most suitable operating points for a trade‐off between the two competing objectives. For rapid optimization of a new process, this approach would be recommended. For more detailed understanding, particularly toward larger scale operation, kinetic, or response surface models are of vital importance. The demonstrated platform provides the flexibility to effectively perform each of these optimization types.

## Conflict of Interest

The authors declare no conflict of interest.

## Supporting information

Supporting Information

## Data Availability

The data that support the findings of this study are available in the supplementary material of this article.
