# Supplementary material for: A Slug Flow Platform with Multiple Process Analytics Facilitates Flexible Reaction Optimization
Source: Adv Sci (Weinh). 2024 Jan 25;11(13):2308034. doi: 10.1002/advs.202308034 (PMC10987115; doi:10.1002/advs.202308034)
Supplement: Supplementary file 1 — Supporting Information [file ADVS-11-2308034-s001.pdf]

ADVANCED SCIENCE
Open Access

Supporting Information

for *Adv. Sci.*, DOI 10.1002/advs.202308034

A Slug Flow Platform with Multiple Process Analytics Facilitates Flexible Reaction Optimization

Florian Wagner, Peter Sagmeister, Clemens E. Jusner, Thomas G. Tampone, Vidhyadhar Manee, Frederic G. Buono, Jason D. Williams and C. Oliver Kappe**

Table of Contents

1. General experimental details....2
2. Control Software 3
3. PAT Instruments 8
4. Determining of Reactor Parameters....15
5. Buchwald-Hartwig Coupling 18
6. Reactor inputs and reactor outputs 20
7. Self-Optimization Experiments....22
8. DoE Experiments 26
9. Kinetic Experiments 32
10. Continuous Flow Validation & Scale-Up Experiments....41
11. Batch Synthesis of Product....44
12. References....45

1. General experimental details

Solvents and chemicals were purchased from commercial suppliers and used without further purification. 1-Bromo-2-nitrobenzene (**ArBr 1**, purity 98%) was obtained from Sigma Aldrich. 2-Amino-5-methylthiophene-3-carbonitrile (**amine 2**, purity 97%) was obtained from Apollo Scientific. $Pd(OAc)_2$ (purity 98%) was obtained from Sigma Aldrich. Xantphos (purity 95%) was obtained from Fluorochem. 1,8-Diazabicyclo[5.4.0]undec-7-ene (**DBU**, purity 98 %) was obtained from TCI. Ethanol (99%) was purchased from VWR. Chlorobenzene (PhCl, purity ≥ 99 %) was purchased from Sigma Aldrich.

Data-Rich Experiments were performed using the automated continuous flow chemistry platform (**Figure S1**, **Figure S2**) at the Kappe Laboratories in Graz. This platform is comprised of a Supervisory Control and Data Acquisition (SCADA) software (Evon, XAMControl), which is further connected to a Distributed Control System (DCS) (HiTec Zang, LabVision software and LabManager hardware), which communicates with actuators and sensors. The platform includes syringe pumps (HiTec Zang, SyrDos2 equipped with high- or low-pressure pump heads) and HPLC pumps (Knauer, AZURA P 4.1S with 10 mL or 50 mL pump heads made out of stainless steel, ceramic or Hastelloy). Additionally, the platform includes thermostats (Huber, Ministat 240 and CC-304), gas and liquid mass flow controllers (Bronkhorst), pressure controllers (Bronkhorst, EL-PRESS) and a hydrogen generator (Thales Nano Energy, H-Genie). A modular micro reaction system (Ehrfeld, MMRS) and a shell-and-tube reactor (Ehrfeld, Miprowa Lab reactor) provide the flexibility of performing different types of reaction in the automated platform. Several real-time PAT instruments such as temperature and pressure sensors, FTIR (Mettler Toledo, ReactIR 15), benchtop NMR (Magritek, Spinsolve Ultra), UV-vis spectrometer (Avantes, AvaSpec ULS2048) and online UHPLC (Shimadzu, Nexera X2) are established within the platform. Automated data processing is accomplished using PEAXACT and ProcessLink (S-PACT), Matlab or Python. Advanced Process Control is enabled by communication to Matlab, Python or DLLs embedded in XAMControl.

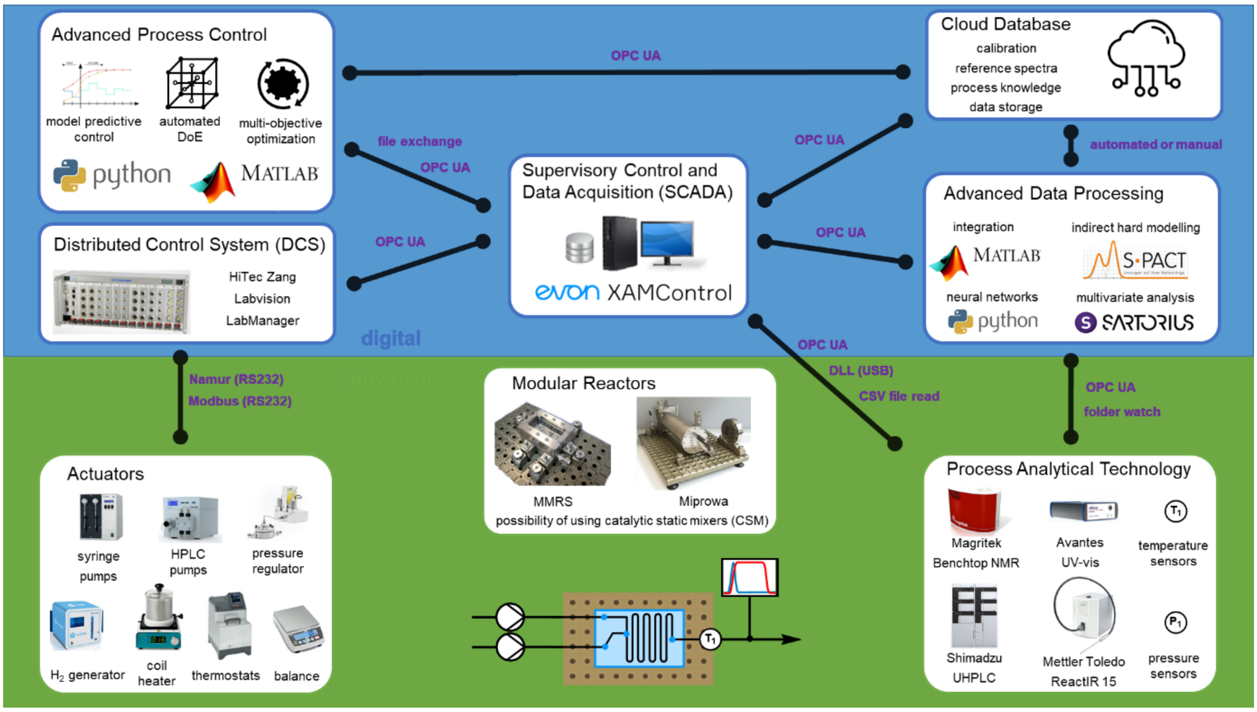

Figure S1 Schematic overview of the automated modular continuous flow platform at the Kappe Lab. Green represents the physical world, blue represents the digital world.

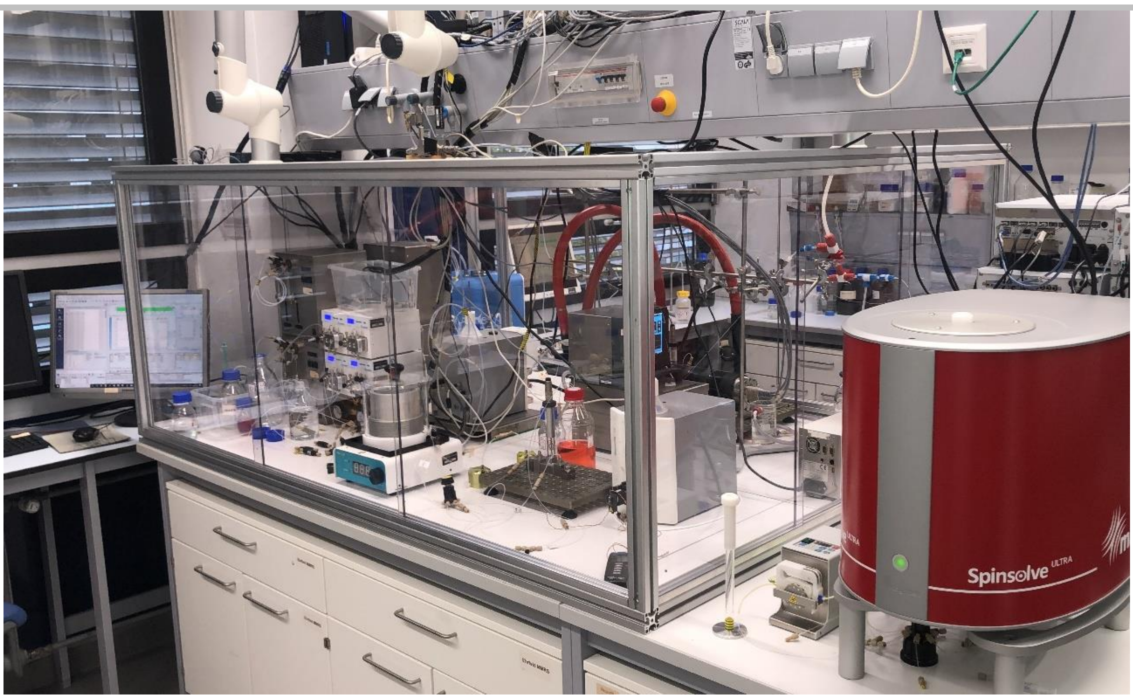

Figure S2. Photograph of the automated modular continuous flow platform at the Kappe Lab.

2. Control Software

XAMControl is a SCADA software designed for industrial automation. It allows for direct communication with actuators and sensors utilizing different field bus protocols as well as OPC UA communication with various DCS systems. The process is visualized using XAMControl Iris (**Figure S4**), which allows for the display of real-time process data and manual process control. XAMControl allows for PLC integration using graphical programming (**Figure S5**) and the C# programming language. All recorded data is stored in a cloud repository and can be accessed using XAMControl Iris or exported as .csv files.

2.1. Process Description

After receiving the start signal, the software reads out a file containing the parameters, those parameters are stored internally by the control software. The control software heats the reactor to the temperature specified in the parameter file. Upon reaching the specified temperature, the setpoints of the pumps are determined, to form a reaction slug in accordance with the ratios specified in the parameter file, with the mixing flow rate (set for the system prior to starting experiments). The control software then calculates the times that the pumps should be turned on for. The pumps are started up and, after a delay to ensure that they have reached the correct flow rate, the signal to inject the first gas bubble is sent. Once the desired amount of reaction mixture has been introduced into the reactor, the software sends the signal to inject the second gas bubble. The software then sets the solvent pump to a flow rate that allows for the desired residence time in the heated reactor to be achieved.
Upon passing through the reactor, the control software switches a VICI 6-port valve to transport the reaction slug to the analytics pathway, first reaching the FTIR (ReactIR 15, Mettler Toledo). The software receives the result of the FTIR detector using a folder watch system (ProcessLink, S-PACT). When the reaction slug is detected by the FTIR, the software waits a defined time period for the reaction mixture to enter the injection valve of the UHPLC (Nexera X2, Shimadzu). Upon entering the injection valve, the software sends a signal that triggers the injection into the UHPLC. After the UHPLC analysis has concluded, the LabSolutions software (Shimadzu) exports the concentration results as a .csv file. This file is read by XAMControl and the concentrations of the analytes are calculated. These concentration values can be processed further.
In the example shown in **Figure S3**, the concentrations are used to calculate the objective values of a self-optimization algorithm, which are then written to an output file. This file is read by a Matlab script and used to calculate the parameters for the next experiment. Matlab then writes the newly calculated parameters to the parameter file to initiate the next experiment.

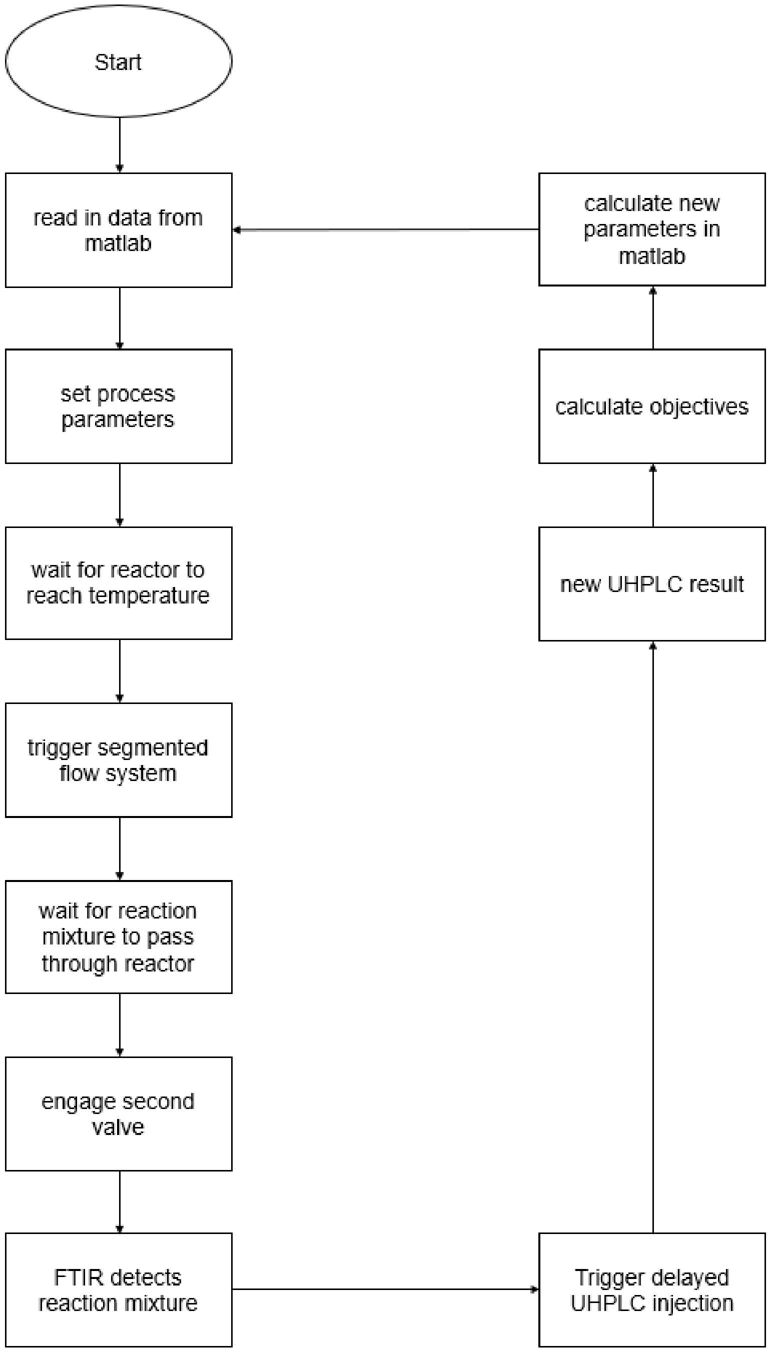

Figure S3. Flowchart of the process implemented in XAMControl.

2.2. Process Visualization

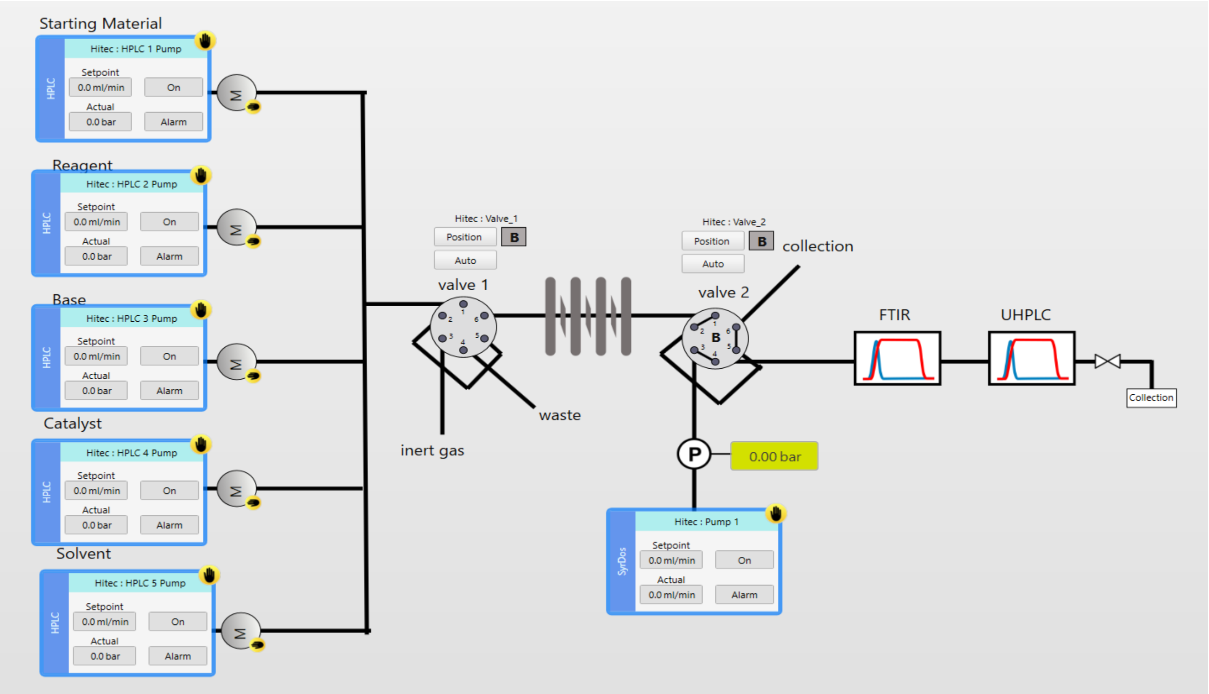

Figure S4 Process visualization in XAMControl for the segmented flow system. The lab equipment and PAT can be monitored and controlled from this view

2.3. Design of the Programmable Logic Controller (PLC)

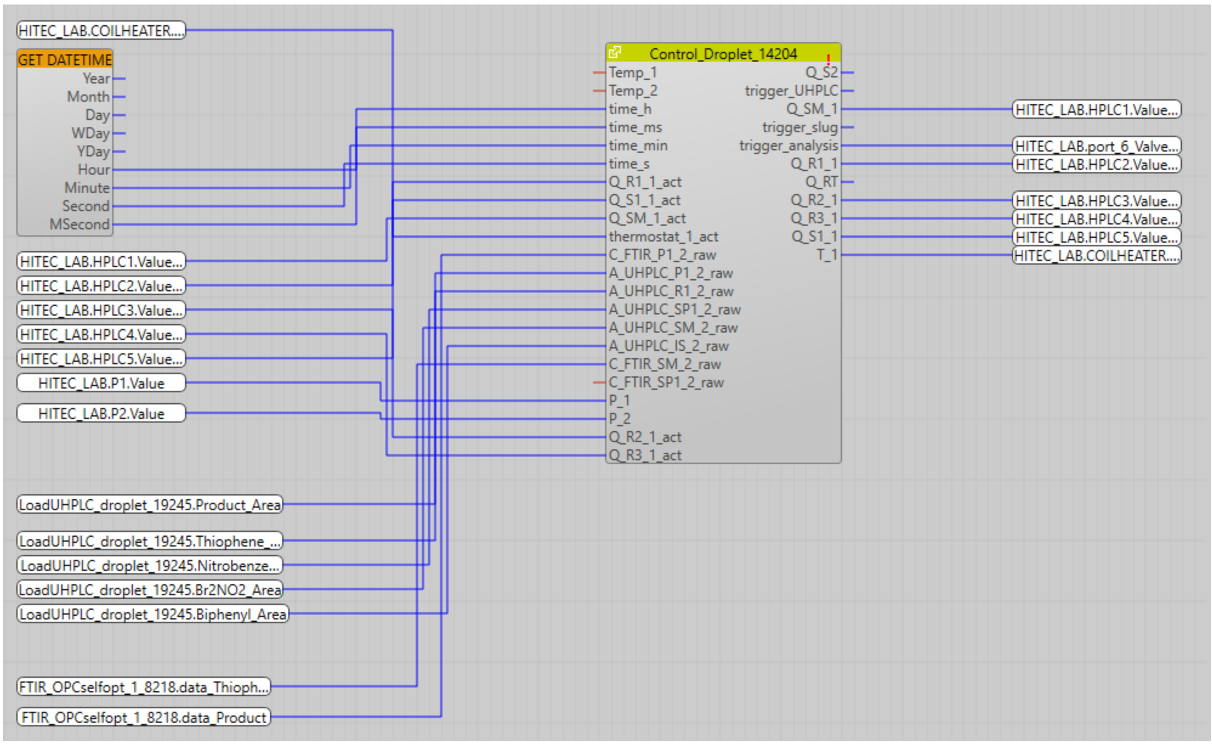

Figure S5. PLC design in XAMControl with the different inputs and outputs of the PLC for the slug flow controller.

2.4. External file handling

To ease the labor-intensive process of building FTIR models using the large amount of data gathered, the data was automatically sorted into folders and tagged with process parameters, reactor outputs and compound results using XAMControl and a Python script according to the flowchart shown in **Figure S6**.

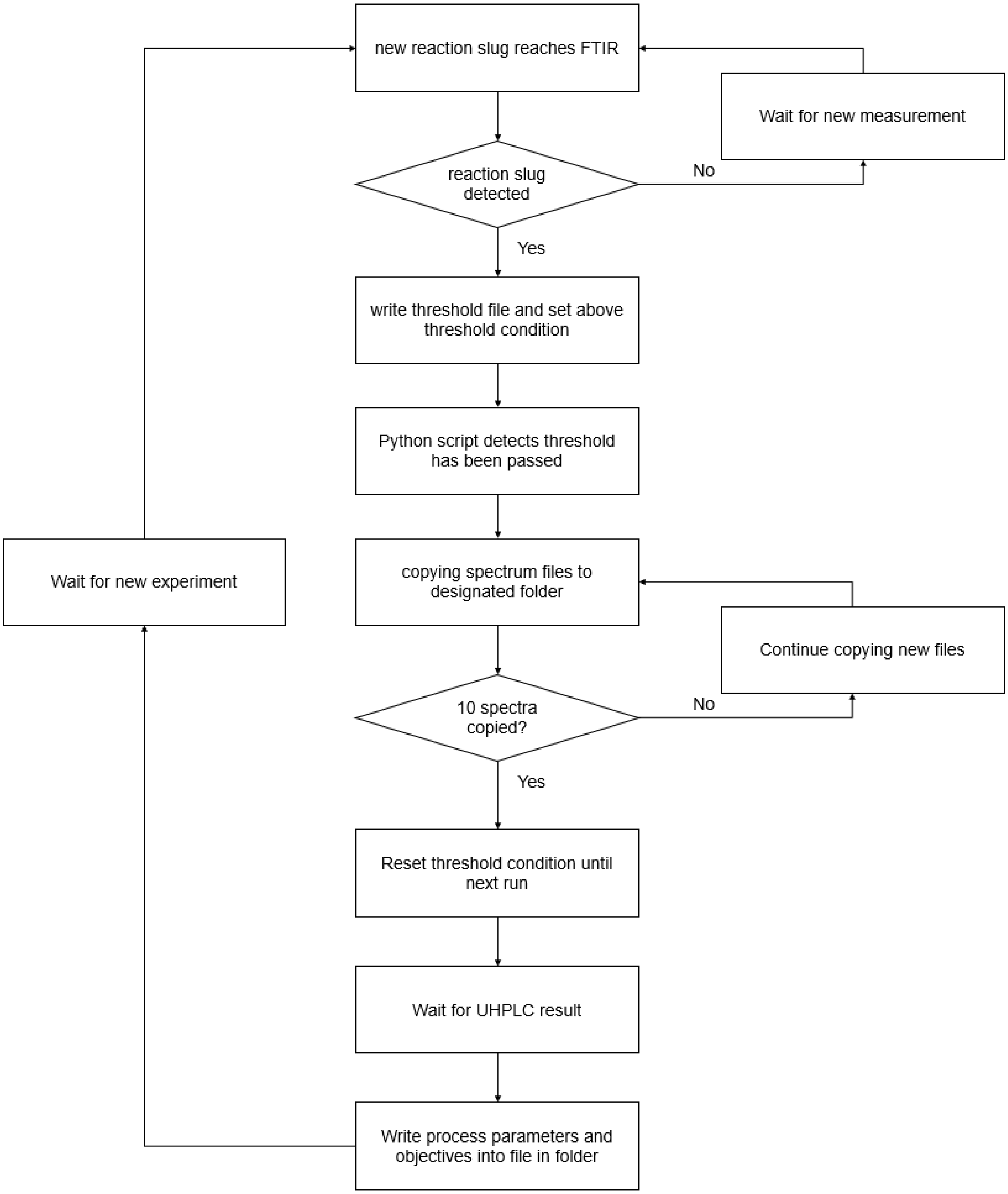

Figure S6. Flowchart of the file handling Python script.

Optimization Algorithm Software

The optimization algorithm TS-EMO was adapted from Schweidtmann et al.[1] with minor changes. The alterations include a file exchange interface between the SCADA software (XAMControl) and the Matlab interface. Further, a Matlab script was developed to automatically generate and execute Latin hypercube sampling. These initial experiments were sorted in order of increasing temperature to reduce the experimental time. This process is shown in **Figure S7**

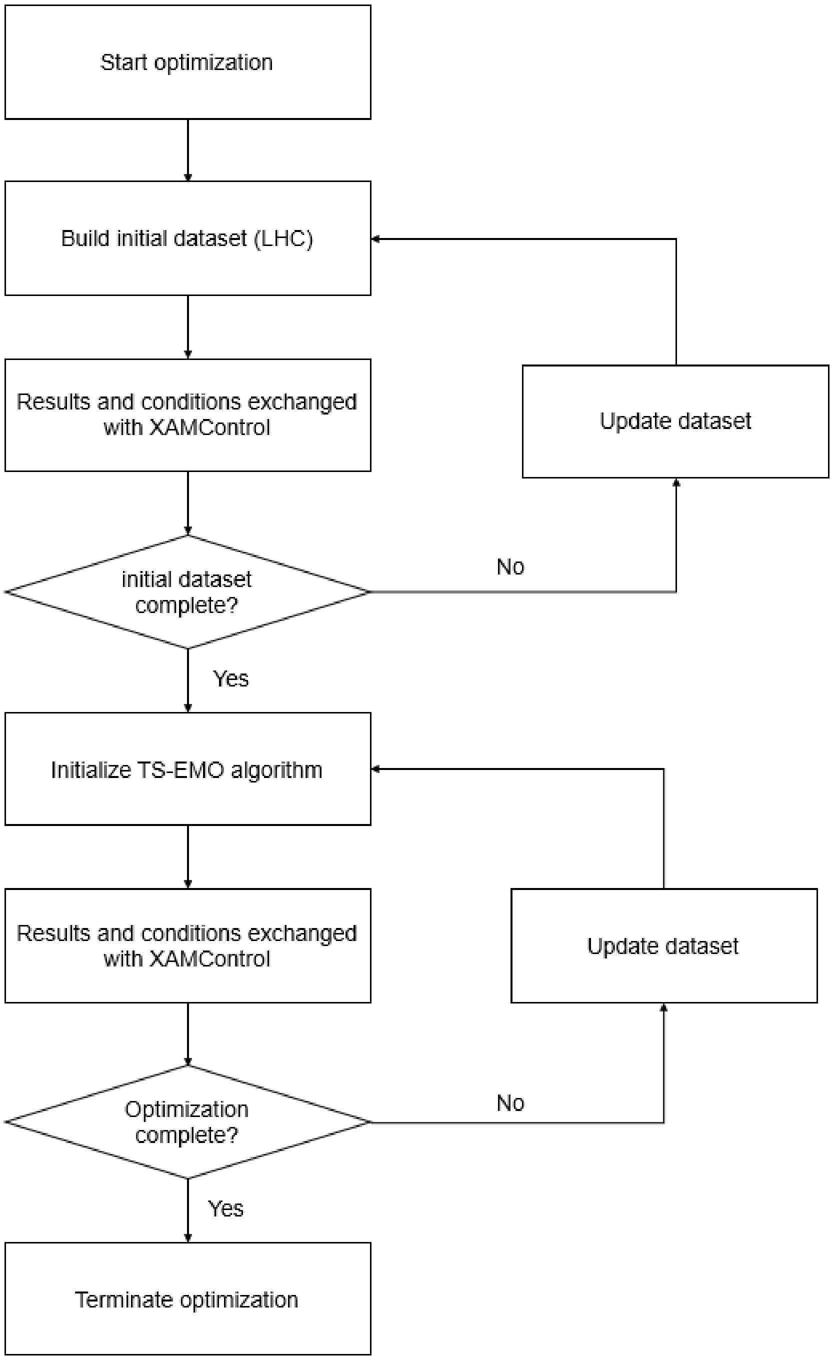

Figure S7. A flowchart of the Self-Optimization algorithm implemented in Matlab.

3. PAT Instruments

3.1. Online UHPLC

General Details and Method:

The UHPLC-DAD (Shimadzu, Nexera X2) was comprised of a degassing unit (DGU-403ASR), two solvent delivery units (LC-30AD), a thermostated column oven (CTO-20AC), a diode array detector (SPD-M30A) and a control unit (CBM-20A). The analysis was carried out using a reversed-phase column (Phenomenex Luna Omega C18 (50 x 2.1 mm, particle size 1.6µm, pore size 100 Å)) at 45 °C using a total flow rate of 1 mL/min. The sample was introduced by an internal injection valve (10 nL, 20000 psi, Cheminert Nanovolume, Part# C84U-6674-.01EUH), which was triggered by the CBM-20A controller. Compounds were eluted with the following gradient: 30% solvent B held for 6 seconds. The amount of B was increased to 50% over 42 seconds, held at 50% for 24 seconds, followed by an increase to 100% over 30 seconds and a final hold at 100% for 30 seconds. The column was then equilibrated at 30% for 48 seconds. The mobile phases A (water/acetonitrile 9+1 v/v +0.1% TFA) and B (acetonitrile + 0.1% TFA) were prepared using HPLC grade ingredients purchased from VWR. Resulting in the chromatogram shown in Figure **S8**.

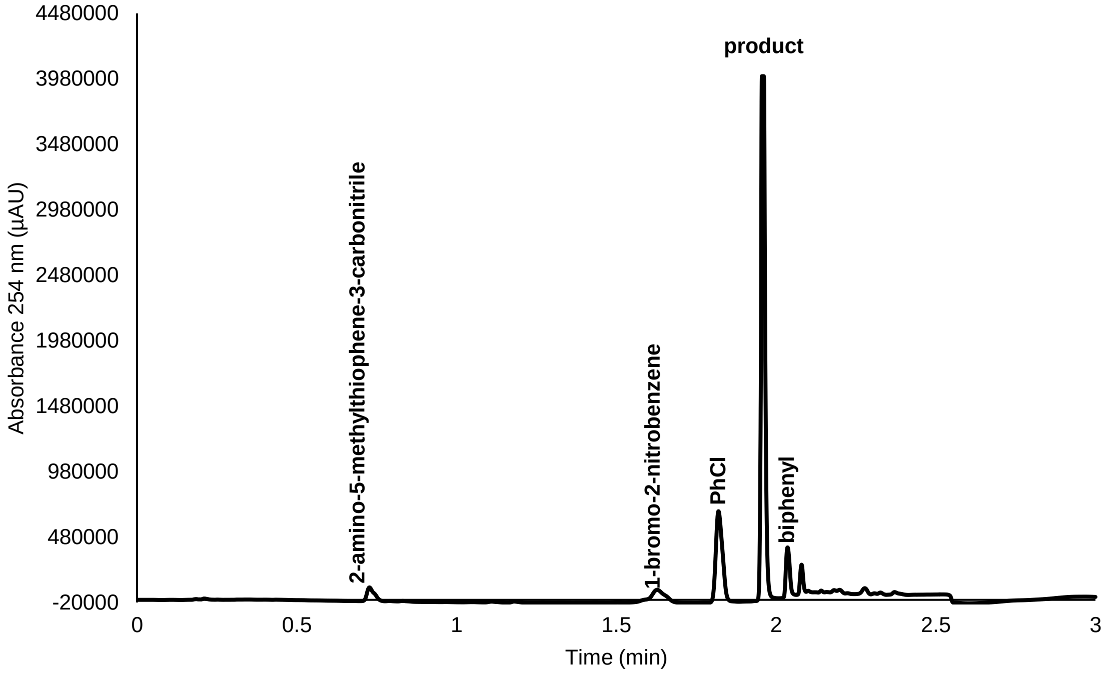

Figure S8 Example chromatogram of the reaction mixture at a wavelength of 254 nm

5-point calibration of the reactants and product against internal standard was carried out using online injections. The internal standard used was biphenyl The calibration curves are shown in **Figure S9**, **Figure S10**, **Figure S11** and **Figure S12**. The anime and aryl halide starting materials were calibrated at 300 nm. The product was calibrated at 341 nm. For biphenyl (internal standard), the absorbance at 254 nm was used.

Table S1. Concentrations of calibration levels in mol/L.

Compounds	Calibration level				
	1 (mol/L)	2 (mol/L)	3 (mol/L)	4 (mol/L)	5 (mol/L)
amine **2**	0.035	0.051	0.10	0.35	0.50
ArBr **1**	0.035	0.051	0.10	0.36	0.50
product **3**	0.035	0.050	0.093	0.34	0.50
nitrobenzene	0.0041	0.0050	0.0086	0.0359	0.0476
biphenyl	0.020	0.020	0.020	0.020	0.020

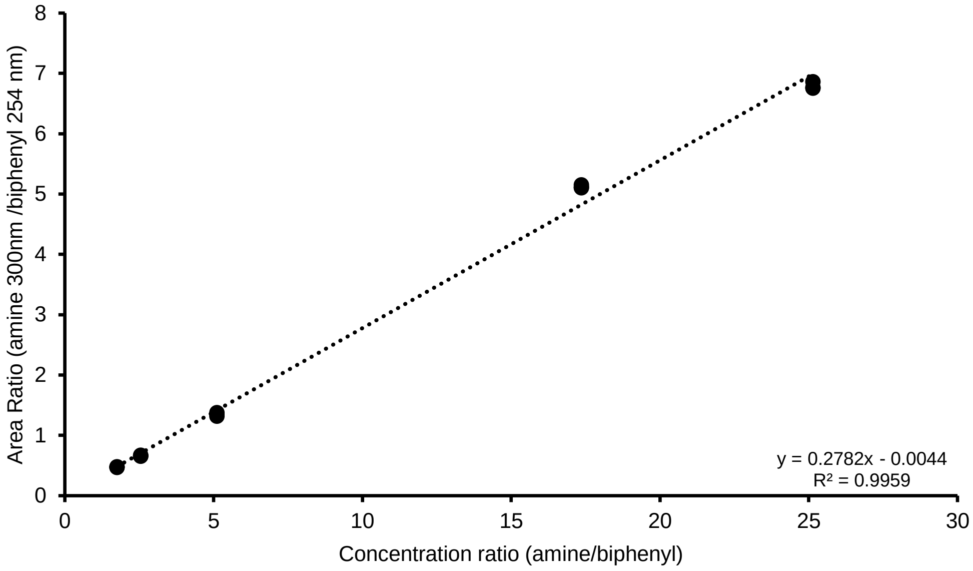

Figure S9. Calibration curve for online UHPLC analysis of 2-amino-5-methylthiophene-3-carbonitrile at a wavelength of 300 nm.

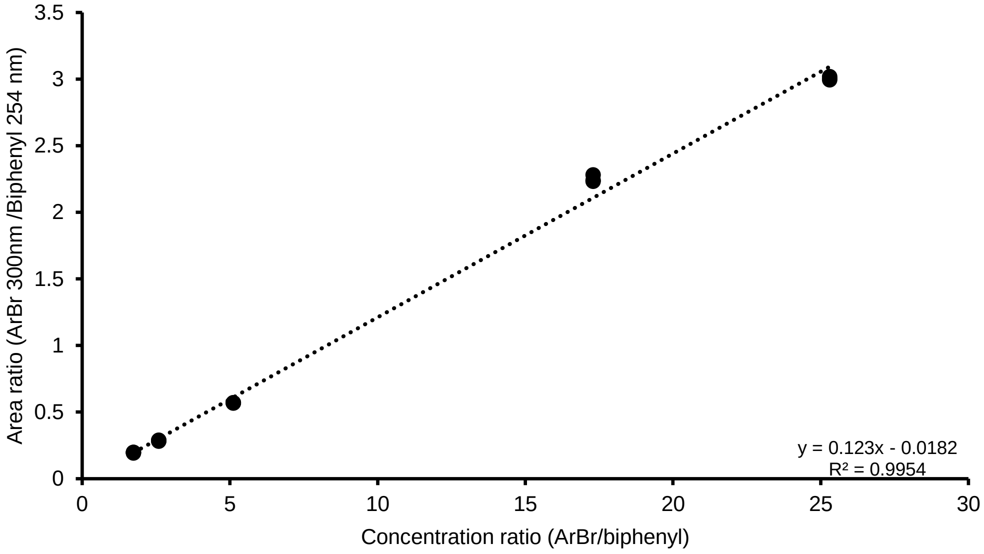

Figure S10. Calibration curve for the online UHPLC analysis of 1-bromo-2-nitrobenzene at a wavelength of 300 nm.

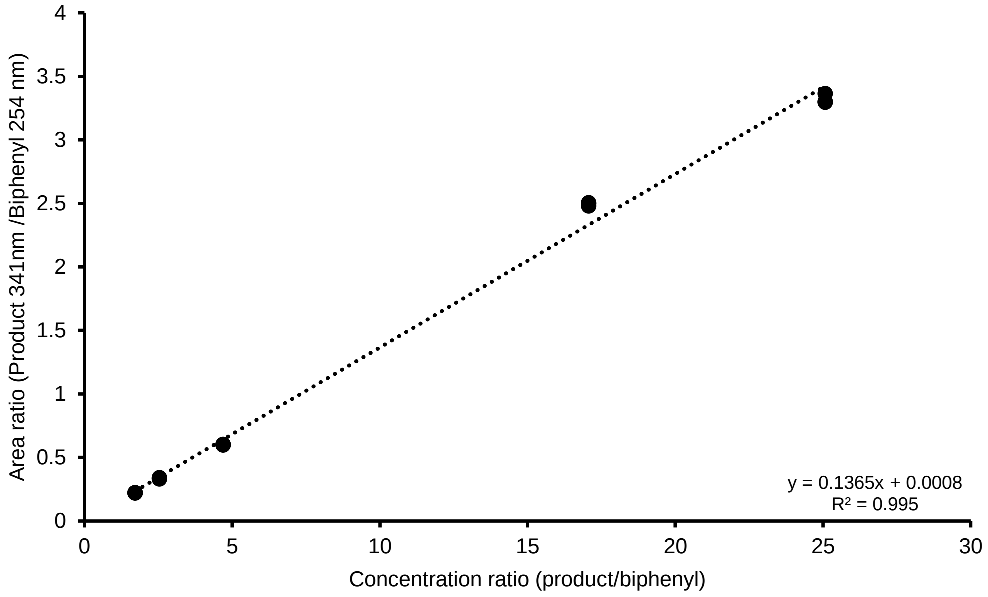

Figure S11 Calibration curve for the online UHPLC of the product at a wavelength of 341 nm

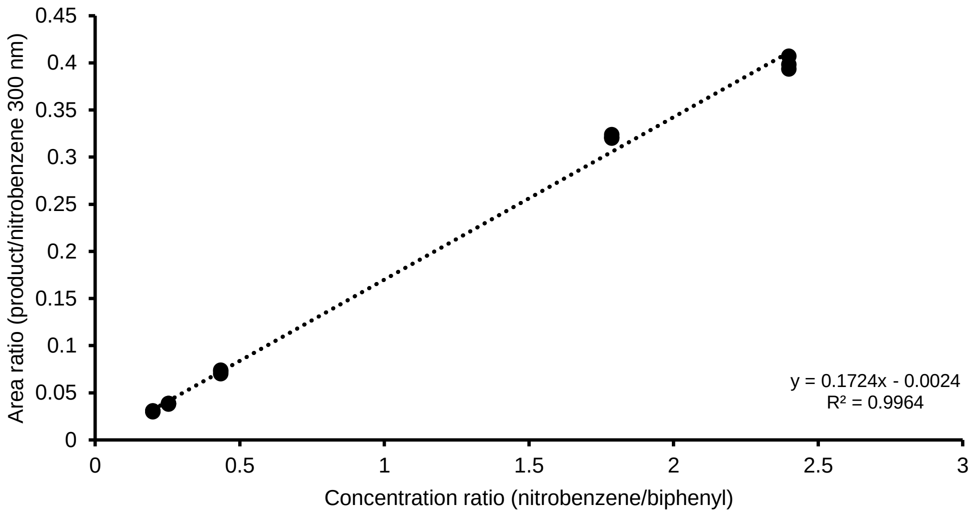

Figure S12. Calibration curve for the online UHLPC of nitrobenzene at a wavelength of 300 nm.

Process integration:
Online UHPLC integration was accomplished using a UHPLC internal sample injector (10 nL, 20000 psi, Cheminert Nanovolume, Part# C84U-6674-.01EUH), controlled using the Shimadzu LabSolutions Software. The chromatographic method engaged the injection valve automatically upon receiving the start signal from the HiTec Zang LabVision software. This start signal was in turn triggered by a processed FTIR measurement.
After each analysis the processed UHPLC data was automatically exported into a .csv file using the Shimadzu LabSolutions software (Version 5.97 SP1) containing information about retention times, areas of analytes and the chromatograms at 254 nm and 300 nm. This file was read by XAMControl and the data contained within was processed further.

Impurities:
A number of impurities have been identified in the process. These impurities are shown in **Figure S13** and **Figure S14**. For the nitrobenzene impurity, the amount was quantified using the calibration curve. The other impurities were quantified as aliquots of the missing mass balance.

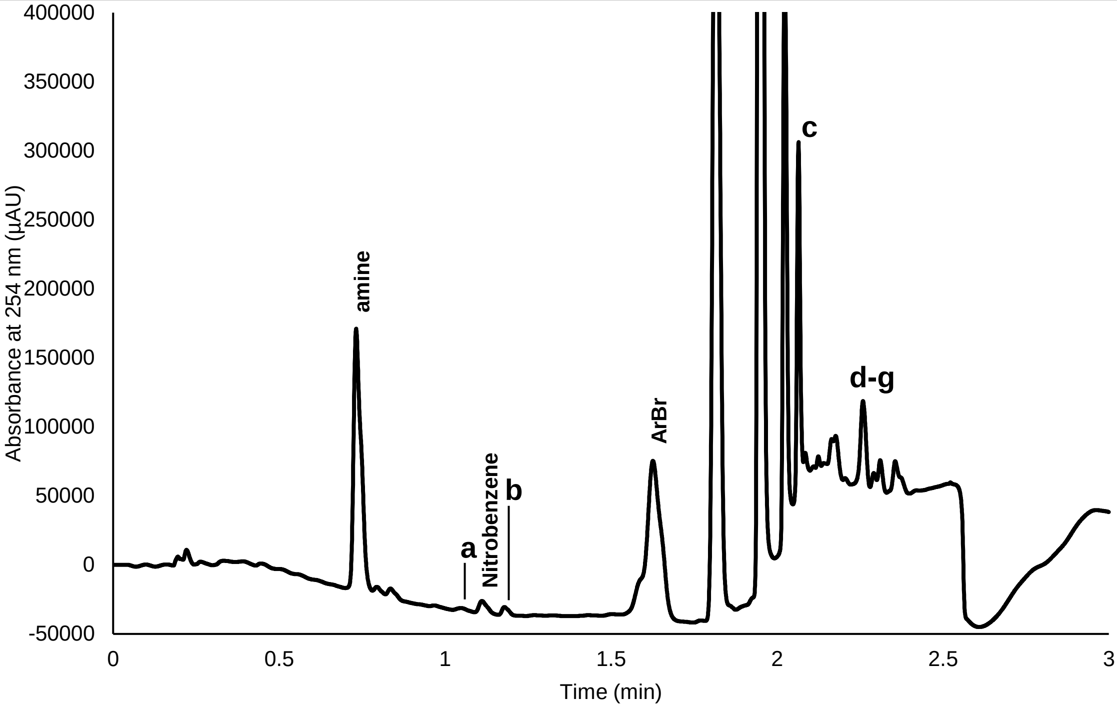

Figure S13. Zoomed-in chromatogram showing minor impurities in the process, unlabeled peaks exceeding plot area are in order of retention time: PhCl, product and biphenyl.

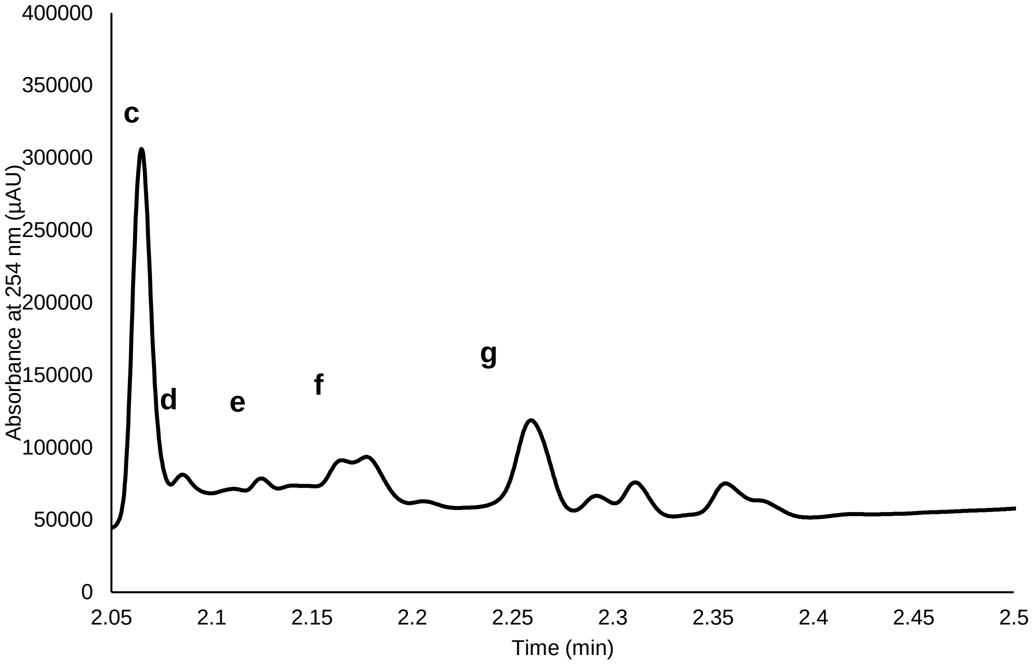

Figure S14. Chromatogram from ***Figure S13*** *zoomed in to focus on region between 2.0-2.5 min.*

3.2. Inline FTIR

General Details:
Inline FTIR Spectra were recorded using a Mettler Toledo ReactIR 15 FTIR (**Figure S15)**, equipped with a flow cell (Mettler Toledo, Micro Flow Cell DS SiComp). The acquisition time was 5 sec per data point and the spectra were recorded between 600 cm^{-1} and 4000 cm^{-1} using a resolution of 4 cm^{-1}. Before use, the MCT detector was allowed to warm to room temperature, then cooled with liquid N_2. It was ensured that the peak height was between 18000 and 24000 and that the signal to noise ratio was above 5000.

Process Integration:
The process stream was connected to the flow cell using a sampling system (**Figure S23**) utilizing a remote-controlled VICI 6-port valve.

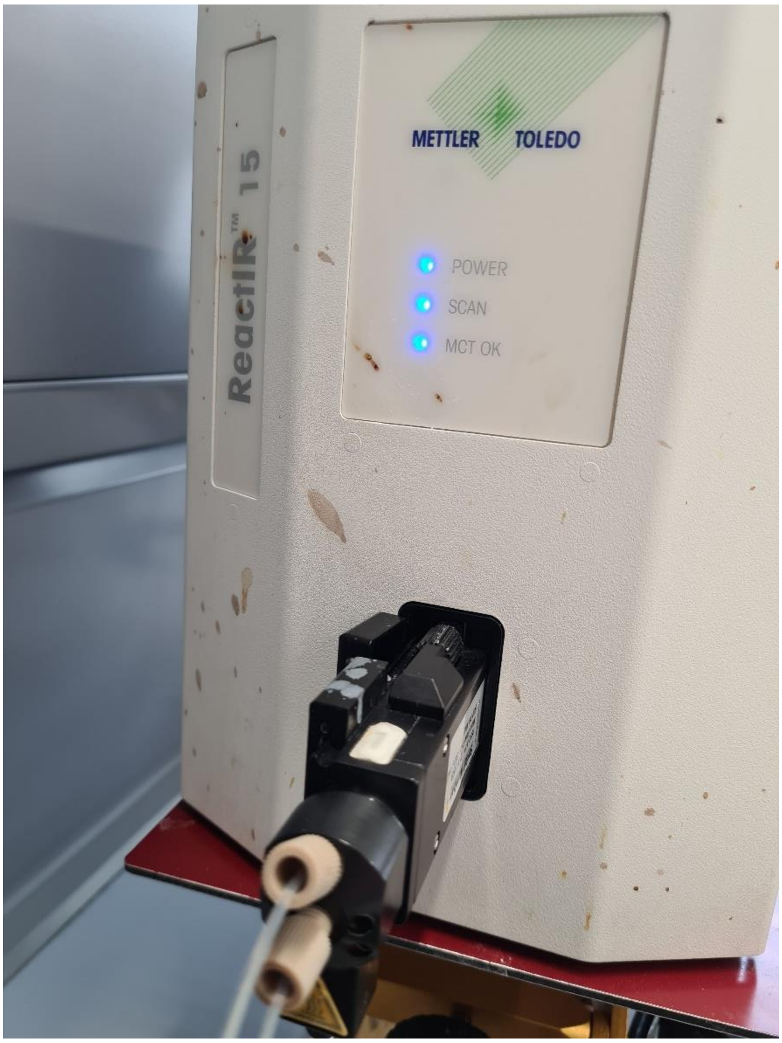

Figure S15. Photograph of ReactIR 15 flow cell setup.

Data processing by integration:
Spectra were pretreated by reducing the wave number range to 2150 cm^{-1} to 2250 cm^{-1} and the whole range was integrated using PEAXACT integration resulting in spectra as shown in **Figure S16**. The resulting integration model was used to determine UHPLC injection timing.

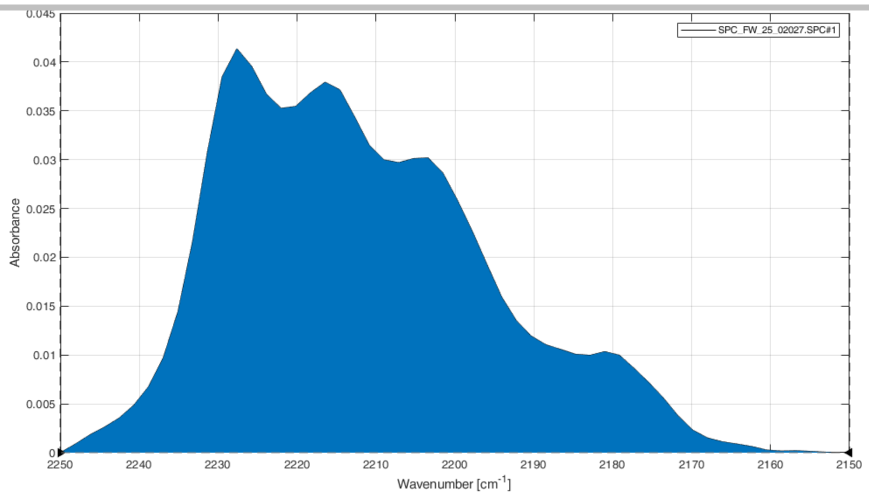

Figure S16. Processed and integrated example FTIR spectrum.

Data Analysis using PLS:

Workflow for PLS regression:
The acquired and pre-sorted spectra from the self-optimization run were loaded into PEAXACT, assigned labels and grouped into different levels.

All spectra underwent the same pretreatment conditions:
Wave number range was reduced to 600 cm^{-1} to 3500 cm^{-1}, rubber band baseline correction, 1st order derivative (filter length = 5) resulting in spectra as shown in **Figure S17**.

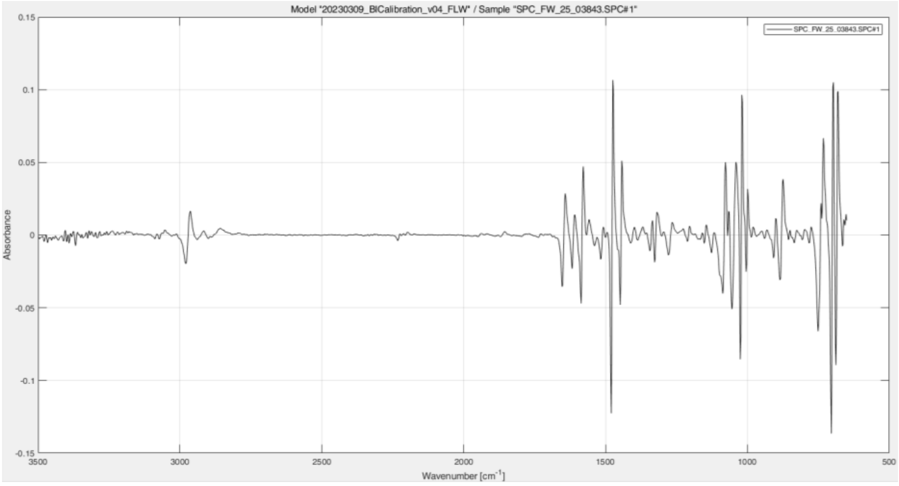

Figure S17. Pre-processed example FTIR spectrum.

Calibration model and validation:
Relevant FTIR spectra from the self-optimization run were collected and assigned labels based on associated UHPLC results, for a total of 47 levels and 569 FTIR spectra. The calibration model provided a performance indicator of model error (root-mean-square error of calibration ($RMSE_C$). Cross-validation was performed using a leave-group-out approach, resulting in the root-mean-square error of cross-validation ($RMSE_{CV}$). The calibration models are shown in **Figure S18**, **Figure S19** and **Figure S20**.To validate the model, the root-mean-square error of prediction ($RMSE_P$) was calculated using the acquired FTIR data from the DoE runs and Kinetic experiments (573 spectra).

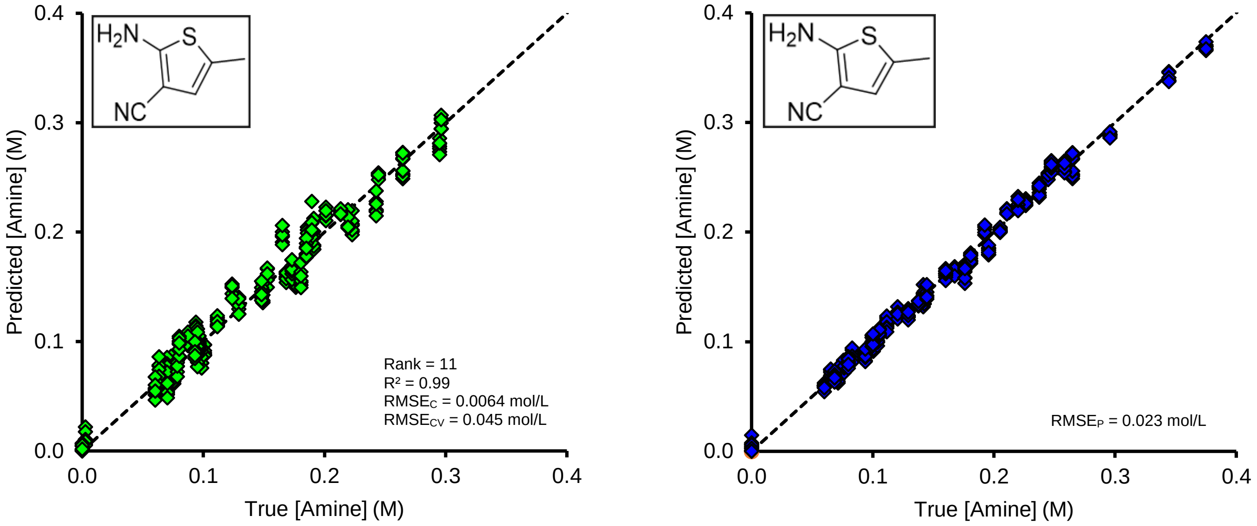

Figure S18. FTIR PLS model Predicted vs. True plot of the amine starting material. green: calibration model, blue: validation data.

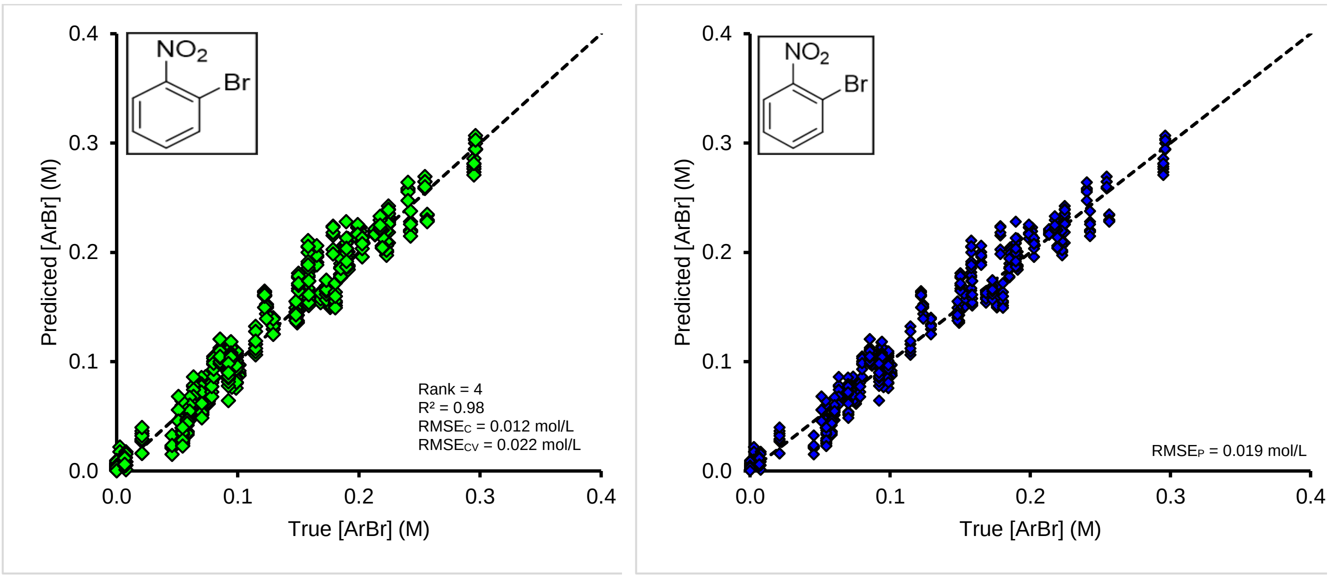

Figure S19. FTIR PLS model Predicted vs. True plot of the ArBr starting material.

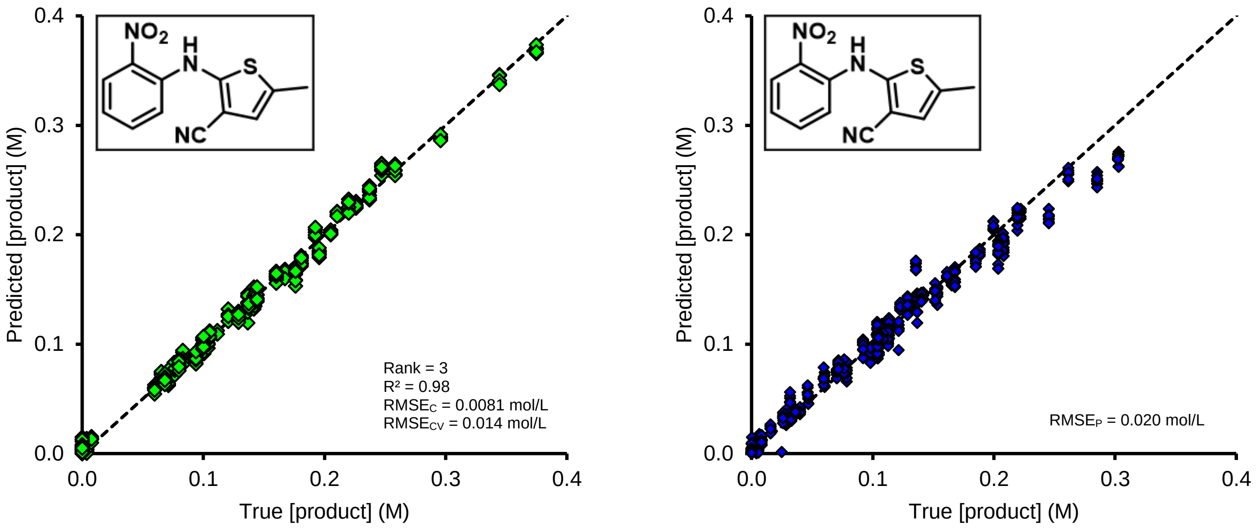

Figure S20. FTIR PLS model Predicted vs. True plot of the product.

4. Determining of Reactor Parameters

To determine viable conditions for the segmented flow regime a range of parameters was examined (**Table S2**). This was carried out according to the following procedure using the setup shown in **Figure S21**:

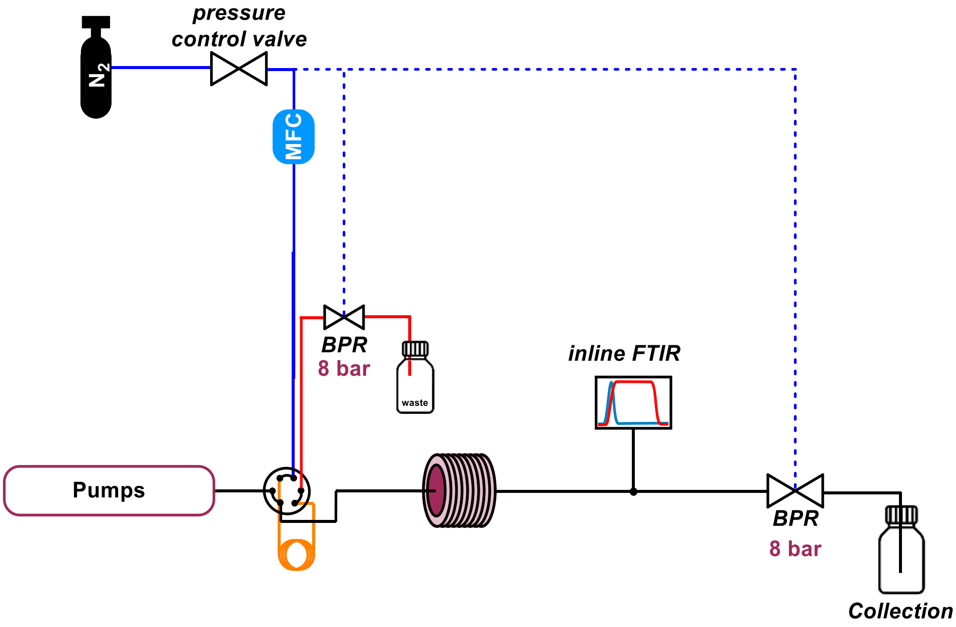

Figure S21. Simplified setup to examine viable volume and flow rate ranges for segmented flow.

Preparation of stock solutions:
1.0 M 1-bromo-2-nitrobenzene solution: 1-bromo-2-nitrobenzene (20.2 g, 100 mmol) was dissolved in EtOH in a 100 mL volumetric flask.
1.0 M Benzophenone solution: Benzophenone (18.2 g, 99.9 mmol) was dissolved in EtOH in a 100 mL volumetric flask.
1.0 M Benzaldehyde solution: Benzaldehyde (10.23 mL, 100,3 mmol) was mixed with EtOH in a 100 mL volumetric flask.

The Knauer AZURA P 4.1S HPLC pumps were charged with the stock solutions and the segmented flow controller was tested using the parameters in **Table S2**. The streams were combined using a 7-way PEEK mixing unit (IDEX P-151, 83 µL i.V.) with one of the ports blocked with a PEEK stopper. The combined streams entered the remote-controlled 6-port valve to form the reaction slug. The

slug then passed through the unheated reactor coil for the specified residence time, before entering the FTIR, the flow rate of the stream was reduced to 0.05 mL/min to allow for a large number of FTIR spectra to be collected. After the FTIR the stream passed through a BPR (Zaiput BPR-10, 8 bar) and into a collection vessel.

Table S2. Conditions tested for segmented flow regime.

Mixing flow rate (mL/min)	0.4	1.0	1.75
Reaction slug volume (µL)	100	300	500
Residence time (min)	5.0	7.5	10.0

The following FTIR traces (**Figure S22**) were obtained by tracking peak height at the following wavenumbers:

Compound:	1-bromo-2-nitrobenzene **1**	benzophenone	benzaldehyde
Wavenumber:	1538 cm^{-1}	1662 cm^{-1}	1707 cm^{-1}

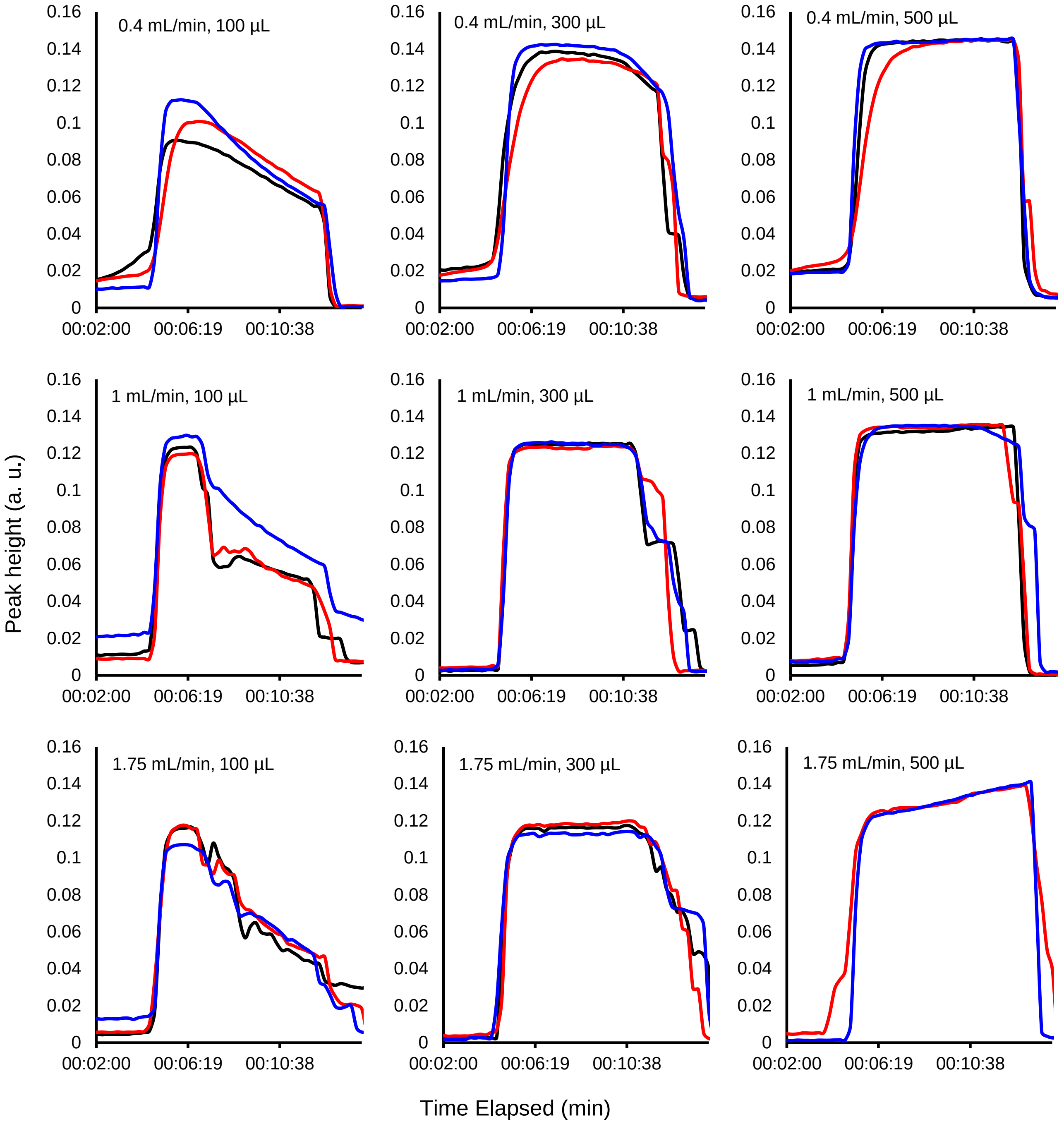

Figure S22. FTIR traces of 1-bromo-2-nitrobenzene at different parameters: black = 5 min t_{res}, red = 7.5 min t_{res}, blue = 10 min t_{res}
Note: 5 min t_{res} unavailable for 1.75 mL/min 500 µL.

5. Buchwald-Hartwig Coupling

5.1. Reactor Platform

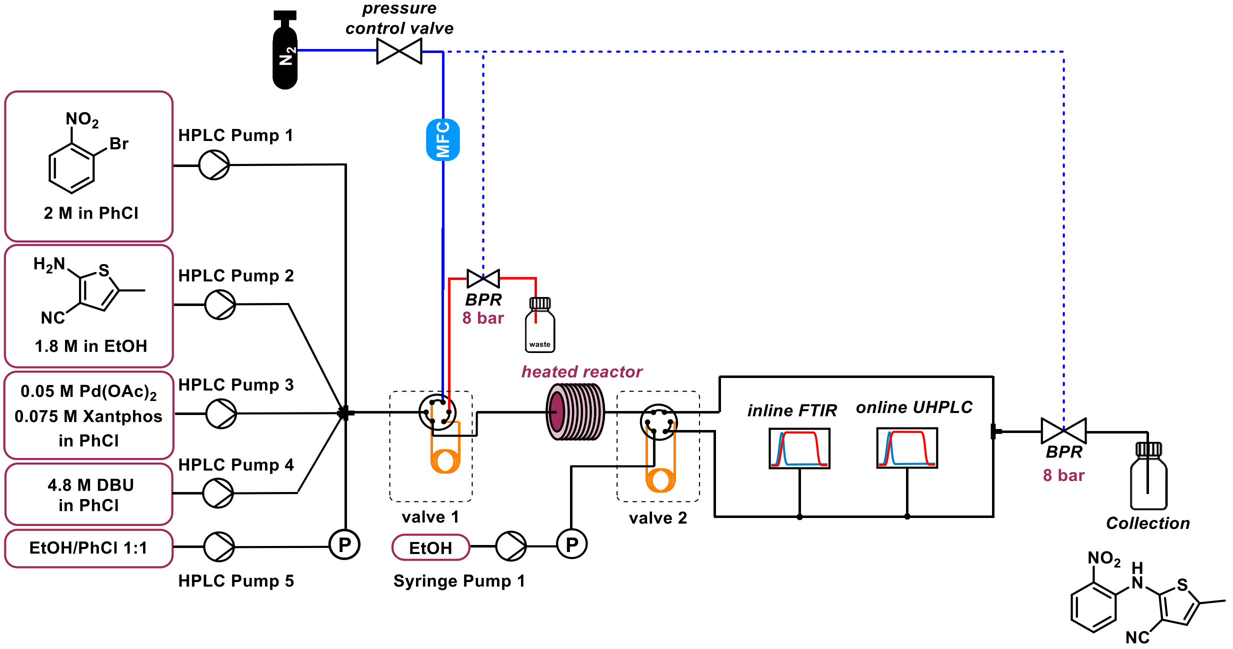

Figure S23. Detailed flow setup of the slug flow reactor platform for the Buchwald-Hartwig coupling towards 3.

The Buchwald-Hartwig coupling was carried out in a Uniqsis coil reactor using 1/16" PFA tubing. The flow scheme is shown in **Figure S23**. Both the solvent feed was equipped with a pressure sensor (Keller, PAA 35XHTC). The feeds were delivered using five Knauer AZURA P 4.1S HPLC pumps. The streams were combined using a 7-way mixing unit (IDEX P-151, 83 µL i.V.) with one of the ports blocked with a PEEK stopper and additionally mixed using a glass bead mixing unit. The combined streams entered the remote-controlled 6-port valve (VICI C2V-2346EUHA) to form the reaction slug. The valve was connected as shown in **Figure S24**. N_2 gas was delivered using a mass flow controller (El-Flow Select, Bronkhorst). The waste port of the valve was connected to a membrane-based BPR (Zaiput, BPR-10) set to 8 bar. After passing through the valve, the reaction slug passed through an unheated spacer of 1/16" PFA tubing (0.51 mL) before entering the coil heater. After passing through the coil heater, the stream entered a second remote-controlled 6-port valve (VICI C2V-2346EUHA). We assumed that the cooling effectively quenched the reaction. The valve was connected as shown in **Figure S25**. The reaction slug entered the loop in valve 2 (position 1) and upon switching the valve to position 1 was transferred to a separate flow system fed by a SyrDos2 syringe pump equipped with a pressure sensor (Keller, PAA 35XHTC). The reaction slug passed through 0.1 mm i.d. PTFE tubing into the flow cell (Mettler Toledo, Micro Flow Cell DS SiComp) of the FTIR (Mettler Toledo, ReactIR 15), then the sample injector of the UHPLC (Shimadzu, Nexera X2). Upon exiting the injection valve, the reaction slug was transported to a membrane-based BPR (Zaiput, BPR-10, set to 8 bars) in 1/16" PFA tubing and collected after passing through the BPR.

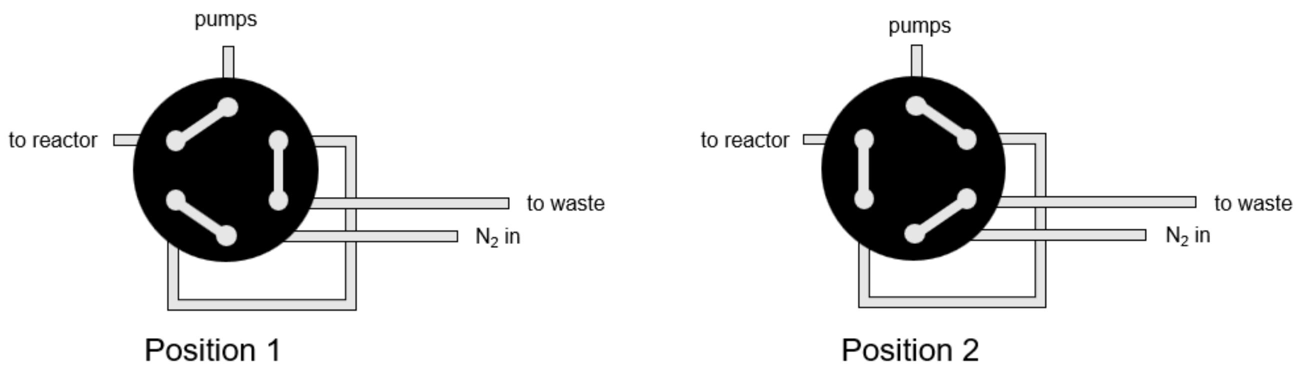

Figure S24. Connectivity of 6-port valve 1.

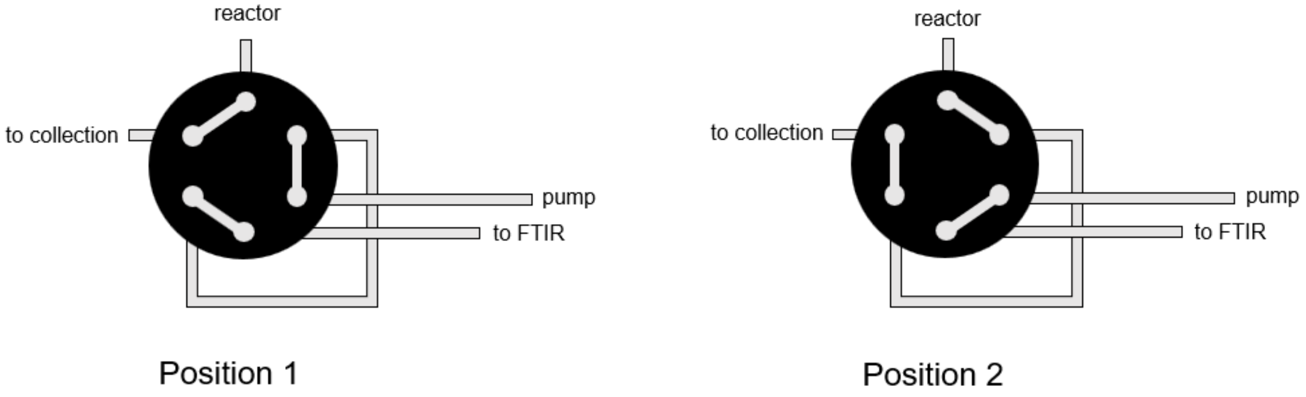

Figure S25. Connectivity of 6-port valve 2.

5.2. Experimental Procedure

Preparation of Stock Solutions:

2.0 M 1-bromo-2-nitrobenzene solution: 1-bromo-2-nitrobenzene (8.0804 g, 40.0 mmol) and biphenyl (308.4 mg, 2 mmol) were dissolved in PhCl in a 20 mL volumetric flask.
1.8 M 2-amino-5-methylthiophene-3-carbonitrile solution: 2-amino-5-methylthiophene-3-carbonitrile (4.975 g, 36.0 mmol) was dissolved in EtOH in a 20 mL volumetric flask.
4.8 M DBU solution: DBU (14.3 mL, 95.8 mmol) was mixed with PhCl in a 20 mL volumetric flask.
0.05 M $Pd(OAc)_2$ solution: $Pd(OAc)_2$ (224.6 mg, 1.00 mmol) and Xantphos (867.9 mg, 1.50 mmol) were dissolved in dry PhCl (10 mL). The solution was degassed with argon for 10 minutes and subsequently handled under argon.
Solvent mixture: In a 250 mL Duran bottle, EtOH (100 mL) and PhCl (100 mL) were mixed (1+1 v/v).

HPLC Pumps were primed with their respective solutions and the slug flow system was initiated. For the self-optimization an implementation of the TS-EMO algorithm, adapted from Schweidtmann et al.,[1] was used. For the DoE/Kinetic experiments a custom batch scheduler written in Python was used.

6. Reactor inputs and reactor outputs

During the self-optimization, DoE and kinetic experiments there is a number of fixed reactor inputs and a number of varied reactor inputs. Additionally, some variables are calculated based on varied inputs. Reactor outputs are measured by UHPLC or FTIR or calculated based on the measured outputs.

The following variables were fixed during experiments:

Initial concentrations:
$\mathbf{C_{SM\_0}}$: concentration of 1-bromo-2-nitrobenzene feed
$\mathbf{C_{R1\_0}}$: concentration of 2-amino-5-methylthiophene-3-carbonitrile feed
$\mathbf{C_{R2\_0}}$: concentration of DBU feed
$\mathbf{C_{R3\_0}}$: concentration of catalyst feed
$\mathbf{C_{IS\_0}}$: concentration of internal standard in 1-bromo-2-nitrobenzene feed

Densities:
$\mathbf{d_{SM\_0}}$: density of 1-bromo-2-nitrobenzene feed
$\mathbf{d_{R1\_0}}$: density of 2-amino-5-methylthiophene-3-carbonitrile feed
$\mathbf{d_{R2\_0}}$: density of DBU feed
$\mathbf{d_{R3\_0}}$: density of catalyst feed

Volumes:
$\mathbf{V_{pre}}$: volume of the sampling section
$\mathbf{V_{1}}$: reactor volume
$\mathbf{V_{post}}$: volume of the loop that transfers reaction slug to the analytics flow system
$\mathbf{V_{to\_UHPLC}}$: volume between FTIR and UHPLC injection valve
$\mathbf{V_{slug}}$: volume of the reaction slug

Costs:
$\mathbf{cost_{SM}}$: calculated cost of 1-bromo-2-nitrobenzene feed in €/mL
$\mathbf{cost_{R1}}$: calculated cost of 2-amino-5-methylthiophene-3-carbonitrile feed in €/mL
$\mathbf{cost_{R2}}$: calculated cost of DBU feed in €/mL
$\mathbf{cost_{R3}}$: calculated of catalyst feed in €/mL

Flow rates:
$\mathbf{Q_{Gas}}$: flow rate of inert gas used to separate slug
$\mathbf{Q_{S2}}$: flow rate of the analytics pump
$\mathbf{Q_{mixing}}$: total flow rate used while making up the reaction slug

Misc.:
$\mathbf{t_{end\_pre}}$: time until reaction slug is fully formed
$\mathbf{P_{BPR}}$: operating pressure of BPRs

The following variables were manipulated during experiments:

$\mathbf{c_{SM0}}$: concentration of 1-bromo-2-nitrobenzene in reaction slug
$\mathbf{r_{R1}}$: Equivalents of 2-amino-5-methylthiophene-3-carbonitrile to 1-bromo-2-nitrobenzene in reaction slug
$\mathbf{r_{R2}}$: Equivalents of DBU to 1-bromo-2-nitrobenzene in reaction slug
$\mathbf{r_{R3}}$: Equivalents of $Pd(OAc)_2$ to 1-bromo-2-nitrobenzene in reaction slug in %
$\mathbf{t_{res}}$: Residence time
T: Temperature

The following variables were calculated based on manipulated inputs during experiments:

Flow rates:
$\mathbf{Q_{SM\_1}}$: flow rate of 1-bromo-2-nitrobenzene feed during mixing
$\mathbf{Q_{R1\_1}}$: flow rate of 2-amino-5-methylthiophene-3-carbonitrile feed during mixing
$\mathbf{Q_{R2\_1}}$: flow rate of DBU feed during mixing
$\mathbf{Q_{R3\_1}}$: flow rate of catalyst feed during mixing
$\mathbf{Q_{S1}}$: flow rate of solvent (EtOH:PhCl, 1:1) feed
$\mathbf{Q_{conv}}$: conversion factor of flow rates to convert total continuous flow rate at t_{res} to total flow rate (Q_{mixing}) for each pump during mixing

Times:
t_{end_post}: time until reaction mixture has fully passed through reactor and entered analytical section
t_{to_UHPLC}: time between FTIR detection and UHPLC injection

The following variables (reactor outputs) were measured during experiments:
C_{SM2_UHPLC}: UHPLC concentration of 1-bromo-2-nitrobenzene
$C_{R1\_2\_UHPLC}$: UHPLC concentration of 2-amino-5-methylthiophene-3-carbonitrile
C_{P_UHPLC}: UHPLC concentration of Product

The following variables (reactor outputs) were calculated during experiments:
Conversion: **conv_UHPLC** = $1 - (C_{SM\_1} / C_{SM\_0})$
Yield: **yield_UHPLC** = (C_P / C_{SM_0})
Selectivity: **selectivity_UHPLC** = yield_UHPLC / conv_UHPLC
Space-Time Yield: **STY_UHPLC** = $(C_{P\_UHPLC} * (V_1 / t_{res}) / V_1 * M_P / 1000 * 60)$
Cost: **cost** = $(cost_{SM} * (0.3 * Q_{SM\_1} * Q_{conv}) + cost_{R1} * (0.3 * Q_{R1\_1} * Q_{conv}) + cost_{R2} * (0.3 * Q_{R2\_1} * Q_{conv}) + cost_{R3} * (0.3 * Q_{R3\_1} * Q_{conv})$

7. Self-Optimization Experiments

During the self-optimization experiments the optimization algorithm could adjust 6 different variables: R1/SM (ratio of reagent 1 (2-amino-5-methylthiophene-3-carbonitrile) to starting material (1-bromo-2-nitrobenzene)), c_SM (concentration of 1-bromo-2-nitrobenzene), t_{res} (residence time in the reactor), T (temperature of the reactor), R_2/SM (ratio of reagent 2 (DBU) to starting material) and catalyst loading (mol % of $Pd(OAc)_2$). The adjustable variables had the following boundaries (**Table S3**). The objective of the self-optimization was to simultaneously maximize the yield and space-time yield, while minimizing the cost as defined by **Equation (1)**.

Table S3 Lower and upper boundaries for the 6 variables varied in the self-optimization experiments

Limits	R_1/SM	c_SM (mol/L)	t_{res} (min)	T (°C)	R_2/SM	catalyst loading (mol %)
Lower	1.07	0.184	2	100	1.07	0.535
Upper	1.5	0.323	10	140	2.14	5.35

minimize [– ln (yield_UHPLC), – ln (STY_UHPLC), ln (cost)] (1)

*Table S4 Results from the self-optimization experiments using a Latin hypercube (LHC) as the initial data set. The adjusted variables were amine (**2**) loading, concentration of ArBr (**1**), residence time in the reactor, reaction temperature, DBU loading and catalyst loading. The objectives were space-time yield, yield and cost.*

Expt Type	Expt #	Loading Amine **2**	Conc **1**	RT	Temp	Loading DBU	Cat. loading	STY	Yield	Cost
		equiv.	(mol/L)	(min)	(°C)	equiv.	(mol%)	(kg/L*h)		(€/Experiment)
LHC	1	1.46	0.22	9.0	100	1.18	1.50%	0.00	0.0%	0.042
LHC	2	1.25	0.26	2.2	105	1.63	3.45%	0.17	9.5%	0.097
LHC	3	1.16	0.20	6.0	108	1.10	3.26%	0.07	13.8%	0.067
LHC	4	1.14	0.30	3.8	113	1.31	5.21%	0.61	51.1%	0.145
LHC	5	1.34	0.23	3.0	115	2.13	1.13%	0.00	0.0%	0.053
LHC	6	1.31	0.24	7.3	118	1.51	2.18%	0.01	1.6%	0.065
LHC	7	1.10	0.28	4.3	120	1.90	1.95%	0.05	5.4%	0.077
LHC	8	1.20	0.28	9.4	124	1.59	4.13%	0.31	67.4%	0.120
LHC	9	1.24	0.25	4.8	128	1.40	4.67%	0.44	54.9%	0.112
LHC	10	1.39	0.32	6.3	131	1.70	0.59%	0.01	1.7%	0.052
LHC	11	1.43	0.19	8.5	137	1.83	2.58%	0.03	8.0%	0.062
LHC	12	1.41	0.31	7.4	137	1.99	4.30%	0.59	90.7%	0.143
TSEMO	13	1.30	0.31	7.8	105	1.38	2.16%	0.00	0.6%	0.080
TSEMO	14	1.22	0.18	7.4	105	1.36	2.30%	0.00	0.1%	0.050
TSEMO	15	1.20	0.21	6.3	120	1.84	4.70%	0.26	50.2%	0.103
TSEMO	16	1.07	0.21	9.9	132	1.18	2.79%	0.07	22.0%	0.062
TSEMO	17	1.33	0.32	9.7	104	1.07	4.00%	0.18	34.9%	0.123
TSEMO	18	1.26	0.21	10.0	140	1.07	4.76%	0.28	86.7%	0.091
TSEMO	19	1.13	0.24	8.4	140	1.07	3.84%	0.26	58.6%	0.087
TSEMO	20	1.29	0.22	2.0	127	1.10	3.55%	0.20	11.5%	0.078
TSEMO	21	1.09	0.27	5.5	126	1.17	3.41%	0.29	37.4%	0.093
TSEMO	22	1.21	0.31	3.9	130	1.11	2.95%	0.28	22.1%	0.095
TSEMO	23	1.24	0.21	2.8	139	1.39	4.20%	0.57	48.6%	0.089
TSEMO	24	1.17	0.28	3.88	140	1.16	0.57%	0.01	0.5%	0.035
TSEMO	25	1.48	0.26	3.97	138	1.23	2.85%	0.13	12.6%	0.079
TSEMO	26	1.50	0.25	5.36	127	1.29	5.06%	0.47	65.2%	0.120
TSEMO	27	1.50	0.21	2.93	132	1.40	4.19%	0.42	37.9%	0.086
TSEMO	28	1.35	0.19	2.04	136	1.07	5.30%	0.64	44.5%	0.091
TSEMO	29	1.09	0.20	3.83	140	1.59	4.03%	0.67	82.5%	0.083
TSEMO	30	1.14	0.18	5.66	140	1.07	4.15%	0.29	56.4%	0.072
TSEMO	31	1.07	0.20	5.45	137	1.10	2.74%	0.11	20.2%	0.056
TSEMO	32	1.30	0.23	4.92	130	1.70	4.10%	0.43	58.5%	0.099
TSEMO	33	1.33	0.20	3.76	139	1.23	5.13%	0.50	61.6%	0.095
TSEMO	34	1.30	0.19	9.81	130	1.07	4.24%	0.16	53.8%	0.076
TSEMO	35	1.07	0.21	4.38	136	1.51	3.83%	0.35	47.4%	0.083
TSEMO	36	1.08	0.22	7.85	140	1.49	2.68%	0.10	23.2%	0.068
TSEMO	37	1.13	0.20	4.61	135	1.64	4.58%	0.38	57.4%	0.092
TSEMO	38	1.11	0.19	4.95	132	1.45	3.25%	0.11	18.7%	0.068
TSEMO	39	1.07	0.20	3.86	139	1.07	3.94%	0.43	62.5%	0.111
TSEMO	40	1.07	0.22	5.95	140	1.07	4.41%	0.22	38.0%	0.091
TSEMO	41	1.08	0.25	6.27	112	1.07	4.10%	0.18	29.1%	0.096

TSEMO	42	1.07	0.19	7.7	138	1.88	4.07%	0.18	46.9%	0.086
TSEMO	43	1.09	0.32	8.9	136	1.12	2.98%	0.19	33.4%	0.099
TSEMO	44	1.18	0.24	6.2	137	1.92	5.35%	0.55	91.1%	0.145
TSEMO	45	1.07	0.18	8.5	137	1.15	5.34%	0.19	57.5%	0.090
TSEMO	46	1.18	0.20	7.2	139	1.47	4.3%	0.33	77.7%	0.086
TSEMO	47	1.12	0.21	2.09	138	1.49	5.3%	0.78	50.7%	0.104
TSEMO	48	1.17	0.21	4.6	129	1.11	2.1%	0.02	3.0%	0.051
TSEMO	49	1.12	0.21	6.46	140	1.38	3.3%	0.27	54.4%	0.073
TSEMO	50	1.14	0.31	2.1	137	1.32	4.0%	1.13	49.7%	0.124
TSEMO	51	1.11	0.32	2.26	122	1.09	4.7%	0.85	38.2%	0.140
TSEMO	52	1.18	0.19	2.00	140	1.18	4.1%	0.47	31.5%	0.077
TSEMO	53	1.50	0.26	4.45	140	1.15	5.2%	0.66	71.4%	0.127
TSEMO	54	1.15	0.26	3.28	139	1.62	3.2%	0.54	43.0%	0.094
TSEMO	55	1.22	0.22	2.25	131	1.07	2.5%	0.06	3.7%	0.059
TSEMO	56	1.29	0.19	5.54	122	1.50	5.3%	0.28	51.8%	0.097
TSEMO	57	1.23	0.31	4.01	136	1.48	4.5%	0.87	70.9%	0.140
TSEMO	58	1.13	0.18	5.89	133	2.01	4.1%	0.24	50.1%	0.083
TSEMO	59	1.23	0.31	4.52	134	1.07	5.3%	0.87	80.5%	0.151
TSEMO	60	1.12	0.26	4.37	127	1.11	5.0%	0.28	30.2%	0.119

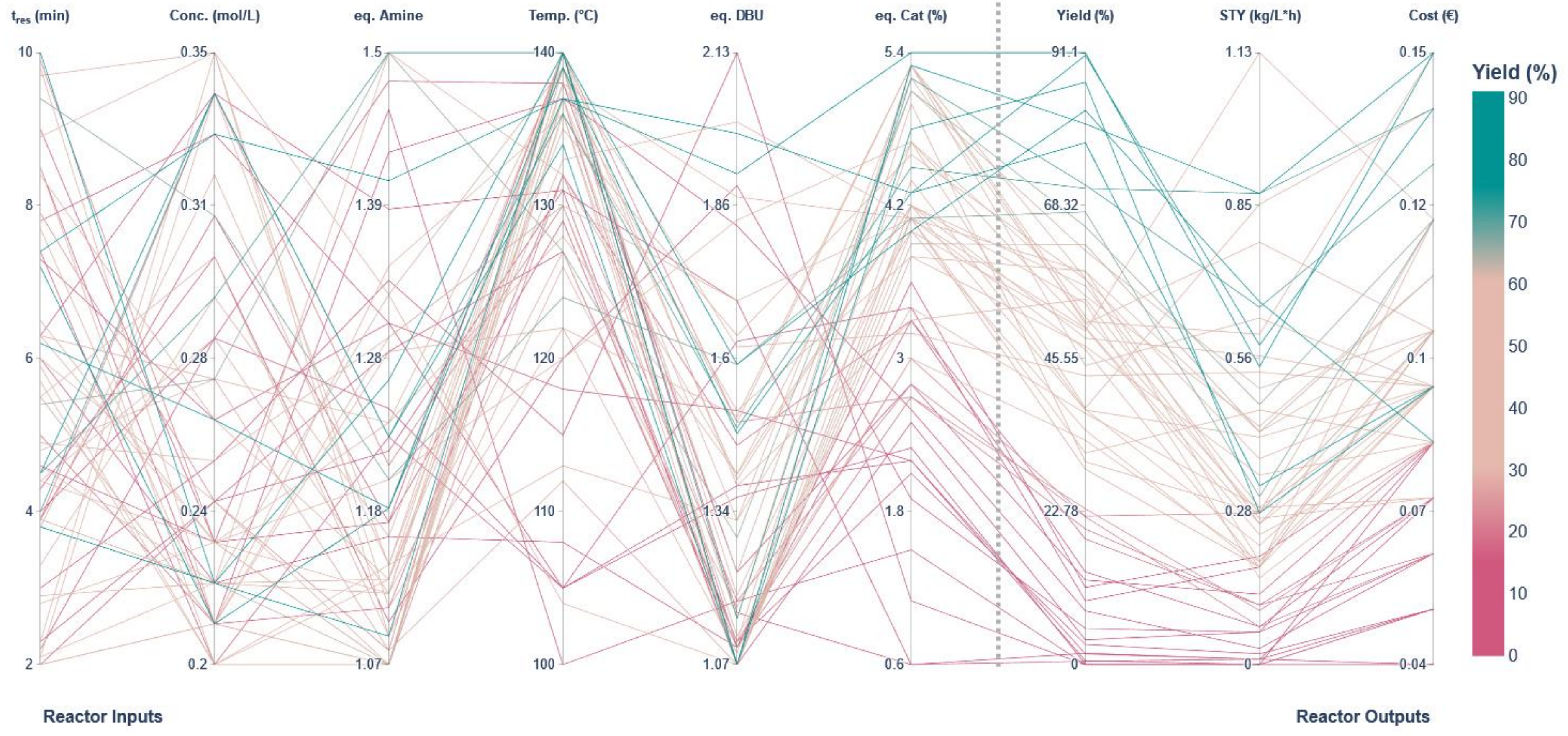

*Figure S26. parallel coordinates plot of self-optimization data, each line represents one entry in Table S4, colors based on product **3** yield (%).*

8. DoE Experiments

During the DoE experiments, four variables were varied to construct a reduced factorial design with additional face points: ratio of reagent 1 (amine) to starting material, temperature of the reactor, ratio of reagent 2 (DBU) to starting material and mol % of reagent 3 ($Pd(OAc)_2$). The concentration of the starting material was fixed at 0.328 mol/L. The residence time was fixed at 6.5 minutes. The boundaries of the adjusted variables are shown in **Table S5**. DoE results are shown in **Table S6** as well as from **Figure S27** to **Figure S31**. Impurities determined during DoE experiments are shown in **Table S7**. The models were fit using main, square and interaction terms.

Table S5. Lower and upper boundaries for the 4 variables varied in the DoE experiments

Limits	R_1/SM	T (°C)	DBU/SM	catalyst loading (mol %)
Lower	1.07	130	1.07	4.28
Upper	1.28	140	1.34	5.35

Table S6. Results from the DoE study performed using the information from the self-optimization to decide on boundary conditions. The adjusted variables were the ratio of amine starting material to ArBr starting material, the temperature of the reactor, the equivalents of base and the mol % of catalyst. Calculated objectives were yield, space-time yield and cost.

	Ratio R_1/SM	Conc_SM (mol/L)	RT (min)	Temp (°C)	DBU/SM	Cat. loading (%)	Yield (%)	STY (kg/L*h)	Cost	c (PhNO2) (mmol/L)
1	1.18	0.328	6.5	135	1.34	4.82%	71.1%	0.56	0.149	3.72
2	1.07	0.328	6.5	130	1.07	4.28%	58.3%	0.46	0.130	3.21
3	1.07	0.328	6.5	130	1.07	5.35%	59.7%	0.47	0.157	2.83
4	1.28	0.328	6.5	130	1.07	5.35%	62.2%	0.49	0.157	3.45
5	1.07	0.328	6.5	130	1.61	4.28%	69.1%	0.54	0.142	4.16
6	1.07	0.328	6.5	130	1.61	5.35%	73.9%	0.58	0.168	3.84
7	1.28	0.328	6.5	130	1.61	4.28%	72.7%	0.57	0.142	4.56
8	1.18	0.328	6.5	130	1.34	4.82%	66.4%	0.52	0.149	3.45
9	1.18	0.328	6.5	135	1.34	4.28%	68.5%	0.54	0.136	4.56
10	1.18	0.328	6.5	135	1.34	5.35%	75.9%	0.60	0.163	4.33
11	1.07	0.328	6.5	135	1.34	4.82%	69.8%	0.55	0.149	4.22
12	1.28	0.328	6.5	135	1.34	4.82%	77.9%	0.61	0.149	4.70
13	1.18	0.328	6.5	135	1.34	4.82%	76.8%	0.60	0.149	4.67
14	1.18	0.328	6.5	135	1.34	4.82%	74.5%	0.58	0.149	4.42
15	1.18	0.328	6.5	135	1.07	4.82%	65.4%	0.51	0.143	4.17
16	1.18	0.328	6.5	135	1.61	4.82%	82.5%	0.65	0.155	5.23
17	1.07	0.328	6.5	140	1.07	4.28%	68.1%	0.53	0.130	4.74
18	1.28	0.328	6.5	140	1.07	4.28%	68.4%	0.54	0.130	5.20
19	1.28	0.328	6.5	140	1.07	5.35%	71.4%	0.56	0.157	4.76
20	1.07	0.328	6.5	140	1.61	5.35%	80.9%	0.64	0.168	5.97
21	1.28	0.328	6.5	140	1.61	4.28%	87.8%	0.69	0.142	6.88
22	1.28	0.328	6.5	140	1.61	5.35%	91.3%	0.72	0.168	5.52
23	1.18	0.328	6.5	140	1.34	4.82%	81.1%	0.64	0.149	5.35
24	1.18	0.328	6.5	135	1.34	4.82%	80.2%	0.61	0.149	4.98
25	1.18	0.328	6.5	135	1.34	4.82%	74.9%	0.59	0.149	4.59
26	1.18	0.313	6.5	135	1.34	4.82%	79.6%	0.6	0.149	3.90
27	1.07	0.313	6.5	140	1.07	5.35%	72.6%	0.54	0.157	3.93

28	1.07	0.313	6.5	140	1.61	4.28%	84.8%	0.64	0.142	5.37
29	1.18	0.313	6.5	135	1.34	4.82%	78.8%	0.59	0.149	4.07
30	1.28	0.313	6.5	130	1.07	4.28%	63.7%	0.48	0.130	3.64
31	1.28	0.313	6.5	130	1.61	5.35%	84.9%	0.64	0.165	3.86

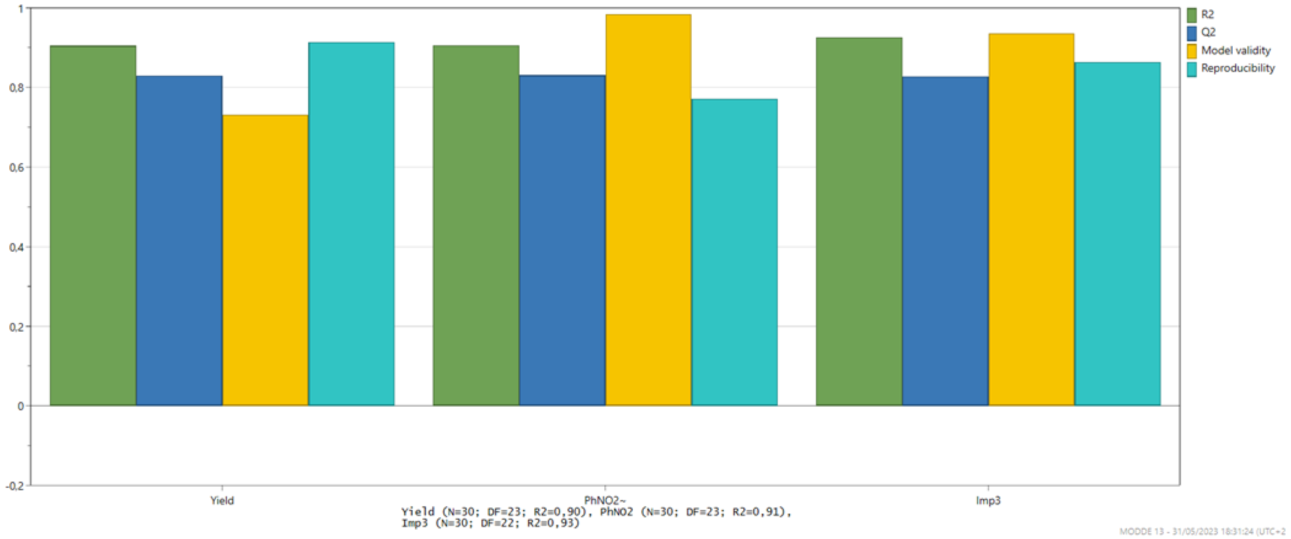

Figure S27. Summary of fit for all models. R^2 is a measure of how well the model fits the data points, Q^2 is a measure of how well the model can predict data which is not part of its initial dataset, Reproducibility is a measure of experimental error.

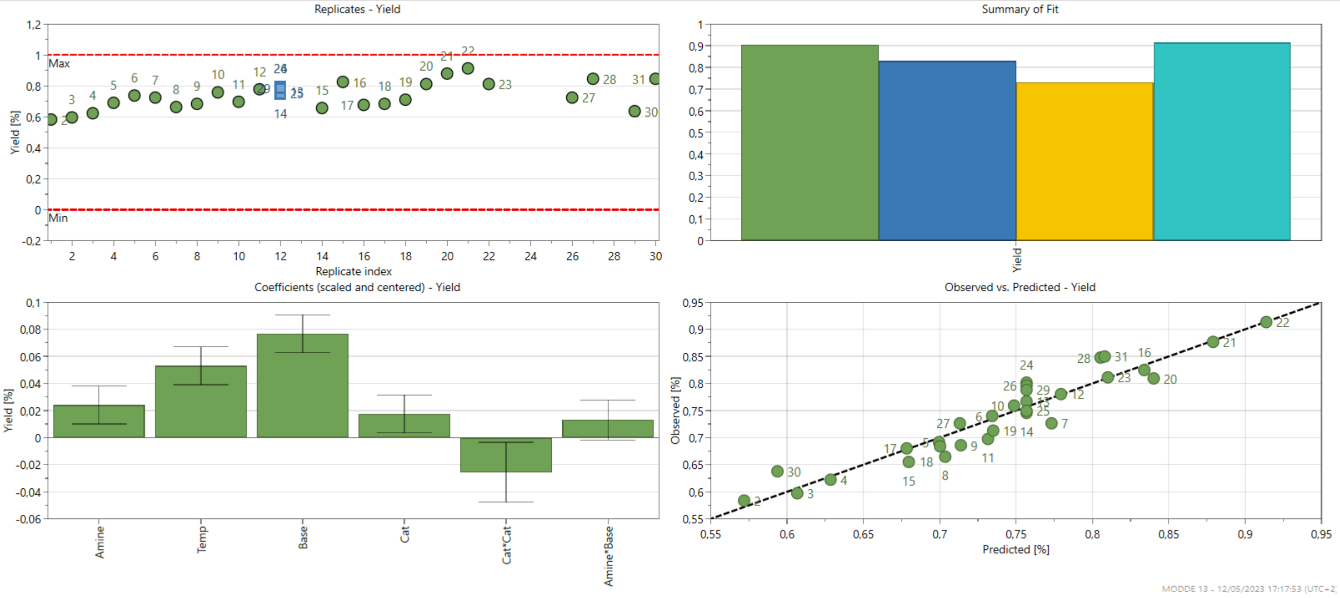

*Figure S28 Summary of the acquired model for the yield of product **3** (response in %). R^2 = 0.90, Q^2 = 0.83, model validity = 0.73, reproducibility = 0.91*

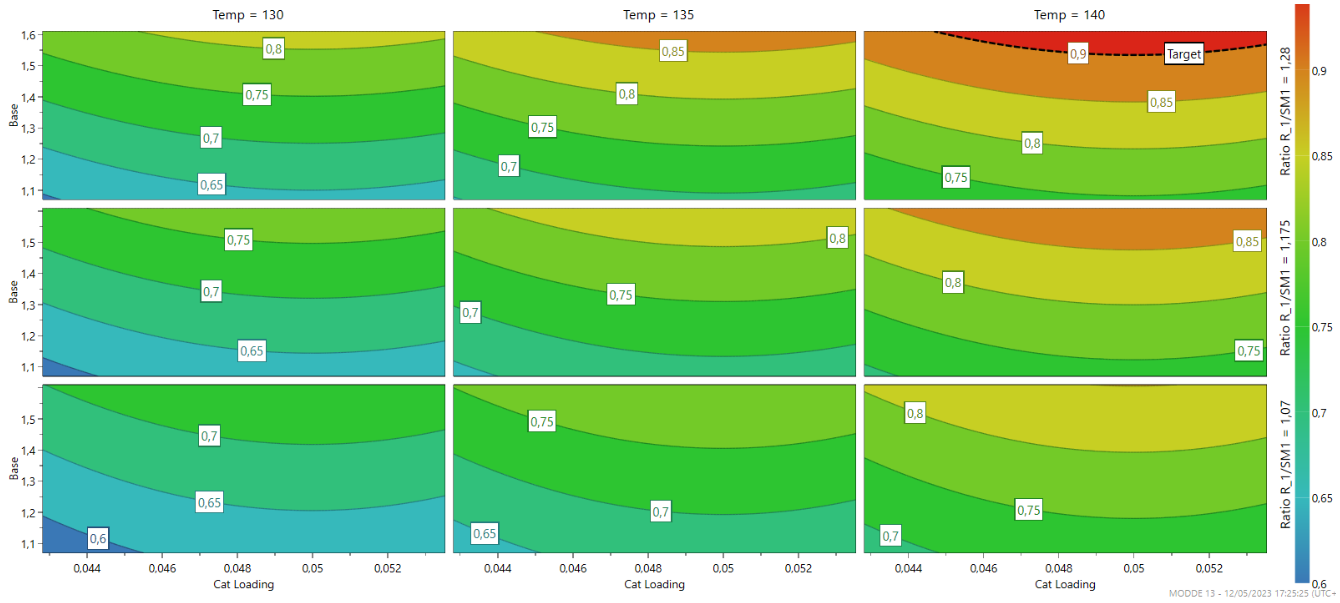

*Figure S29 Response contour plot for the yield of product **3** (response in %).*

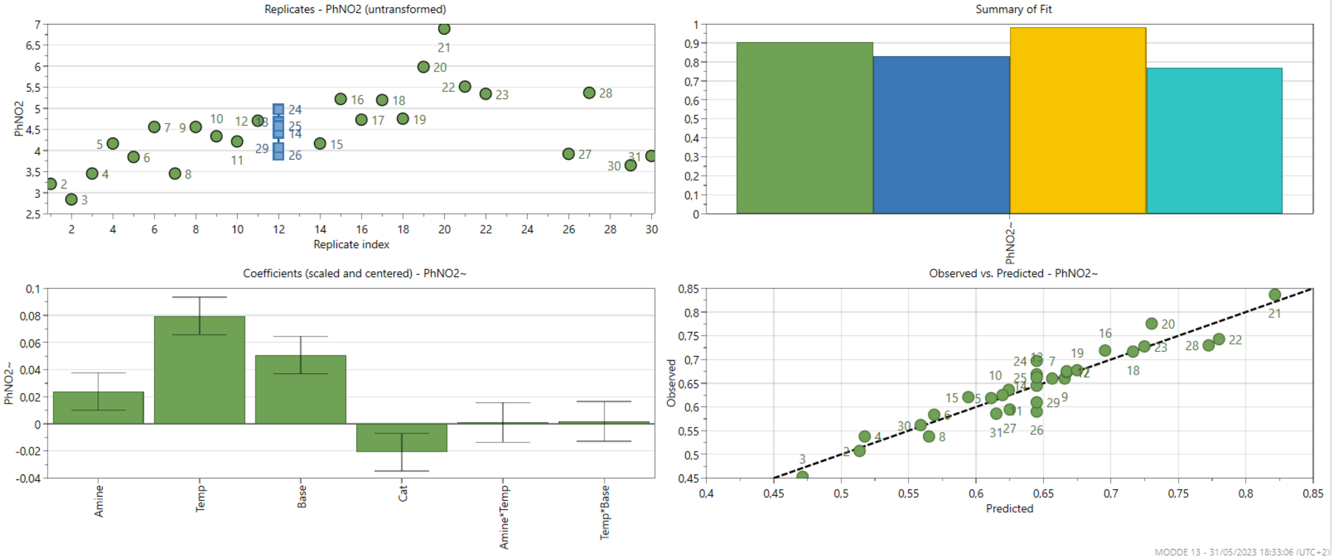

Figure S30. Summary of the acquired model for the nitrobenzene impurity (response in mM). $R^2 = 0.91$, $Q^2 = 0.83$, model validity = 0.98, reproducibility = 0.77.

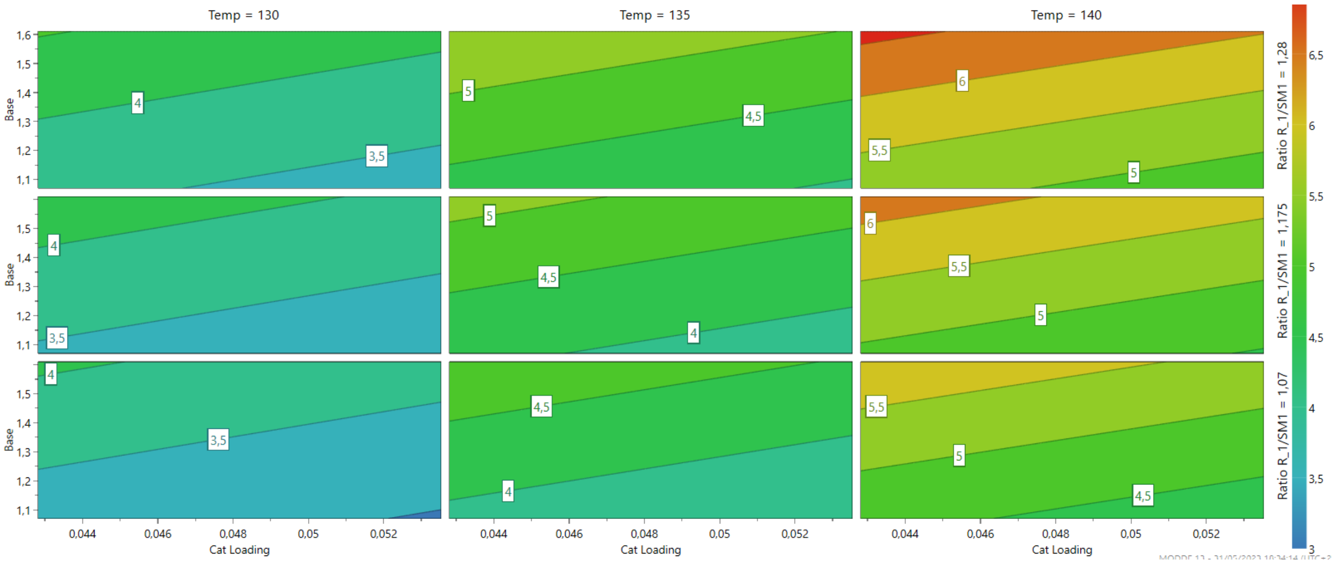

Figure S31. Response contour plot of the nitrobenzene impurity (response in mM).

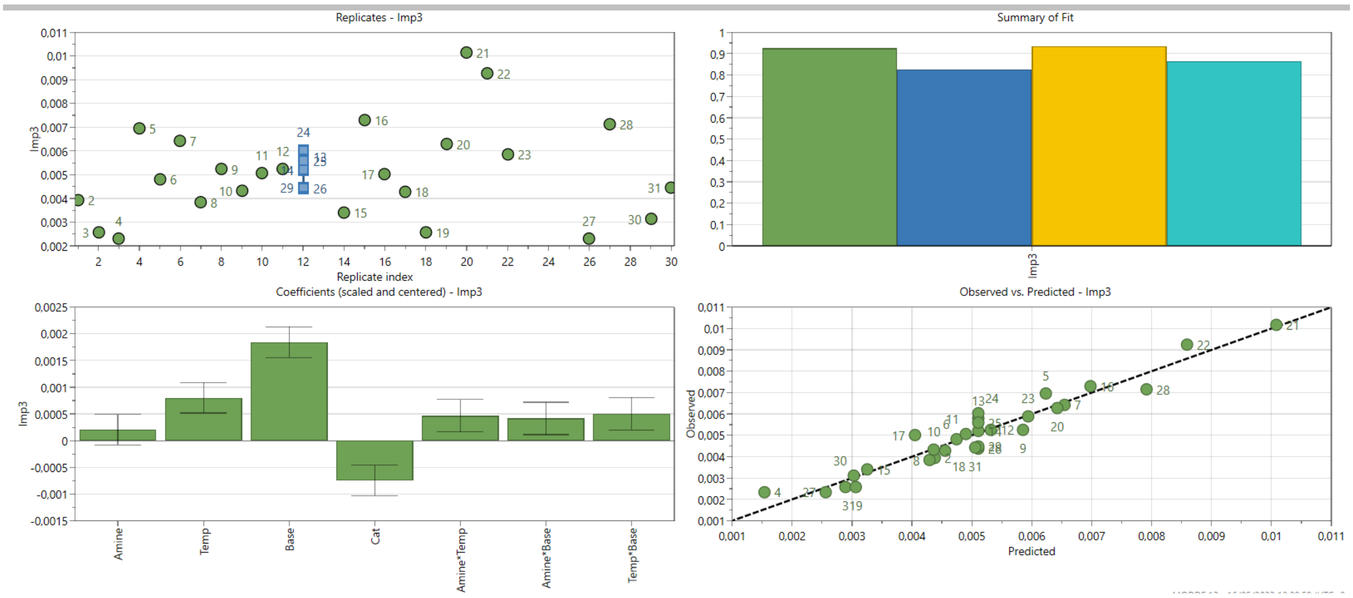

Figure S32. Summary of the acquired model for unidentified impurity ***b*** *(response as fraction of total HPLC area (area% / 100).* $R^2 = 0.93$, $Q^2 = 0.83$, *model validity = 0.94, reproducibility = 0.86.*

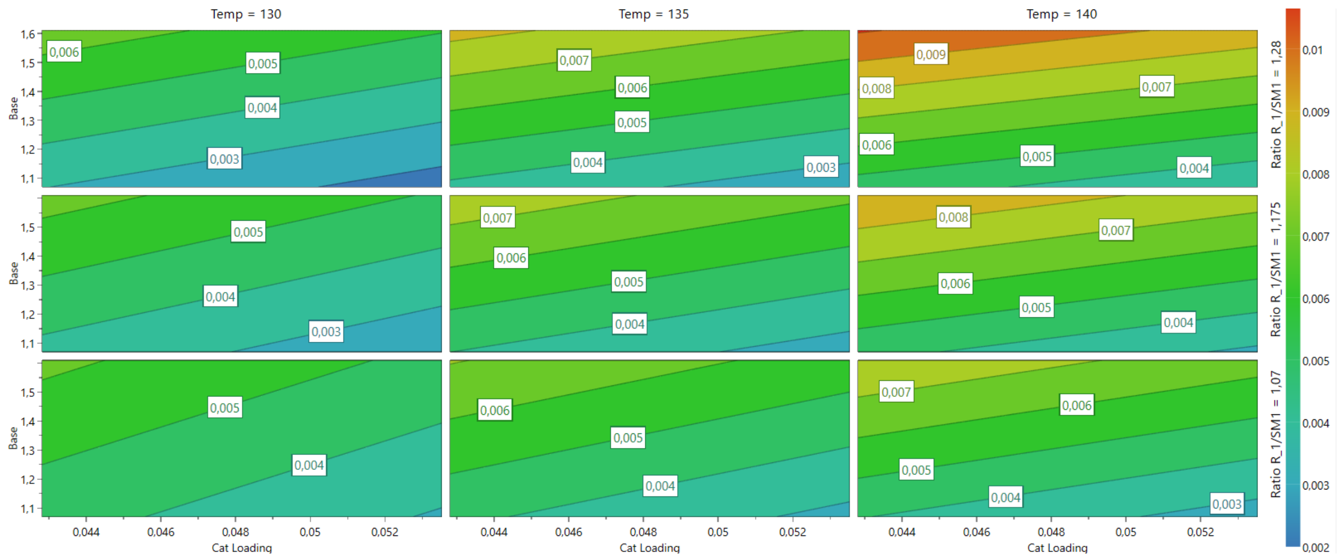

Figure S33. Response surface plot for unidentified impurity ***b*** *(response as fraction of total HPLC area (area% / 100).*

Table S7 Unidentified impurities in DoE experiment series in area % (only considering unidentified impurities) areas at 254 nm, as well as the associated missing mass balance for that experiment.

	Missing mass balance	Impurity **a**	Impurity **b**	Impurity **c**	Impurity **d**	Impurity **e**	Impurity **f**	Impurity **g**
	(mmol/L)	(Impurity area %)	(Impurity area %)	(Impurity area %)	(Impurity area %)	(Impurity area %)	(Impurity area %)	(Impurity area %)
1	27.84	3	5	45	4	14	19	9
2	11.51	2	4	44	4	4	27	15
3	35.93	2	3	50	4	6	22	14
4	29.60	1	3	44	4	4	28	16
5	29.22	3	8	48	2	7	22	11
6	29.76	2	5	53	9	6	16	9
7	20.64	3	7	42	5	4	27	13
8	29.82	2	4	47	6	4	23	13
9	26.00	2	6	47	2	5	26	12
10	28.75	2	5	54	2	5	21	12
11	32.63	2	5	56	2	4	20	11
12	25.04	2	6	49	1	4	25	12
13	29.56	2	6	51	2	5	23	11
14	27.15	2	6	51	1	4	24	11
15	30.27	2	4	51	2	4	25	13
16	29.20	3	8	52	1	5	21	9
17	29.88	2	6	54	1	4	21	12
18	26.63	2	5	48	0	3	28	14
19	29.60	1	3	55	1	2	25	12
20	24.76	3	7	63	1	3	16	7
21	28.63	4	12	41	0	4	28	11
22	20.76	13	10	15	1	7	34	18
23	25.09	2	6	56	1	3	21	10
24	21.86	2	7	52	1	5	23	10
25	28.36	2	6	52	1	5	23	11
26	8.80	2	5	49	2	7	26	9
27	12.43	1	2	57	1	5	23	10
28	9.35	3	8	51	1	5	25	7
29	7.43	2	5	52	1	7	25	8
30	55.33	1	3	40	2	8	34	11
31	4.60	2	5	46	1	10	25	10

9. Kinetic Experiments

During the kinetic experiments six sets of conditions (**Table S8**) were examined at residence times of 0.5 min, 1 min, 2 min, 4 min, 8 min, 12 min:

Table S8. Conditions used in kinetic experiments.

Experiment series	R_1/SM	c_SM (mol/L)	T (°C)	R_2/SM	catalyst loading (mol %)
1	1.30	0.323	140	1.63	5.11
2	1.30	0.323	140	0.82	5.11
3	1.09	0.231	125	1.63	5.43
4	1.09	0.231	130	1.63	2.72
5	0.87	0.268	135	1.09	4.89
6	1.20	0.268	130	1.63	3.26

The following kinetic profiles were obtained (**Figure S34** to **Figure S39**):

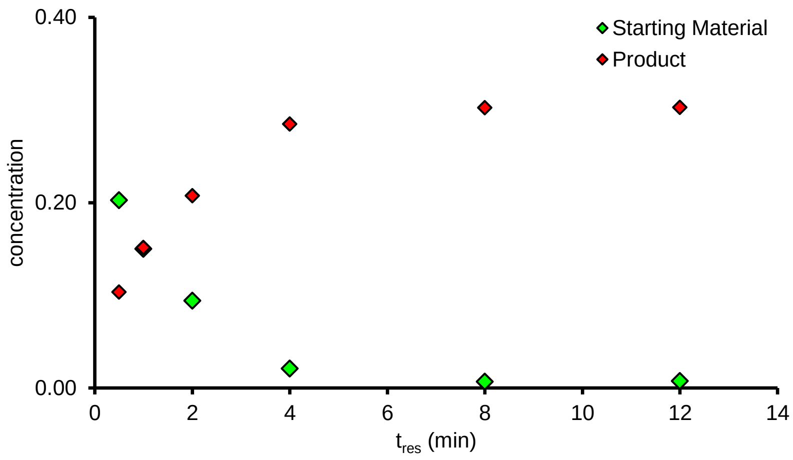

Figure S34. Time course data obtained from experiment series 1.

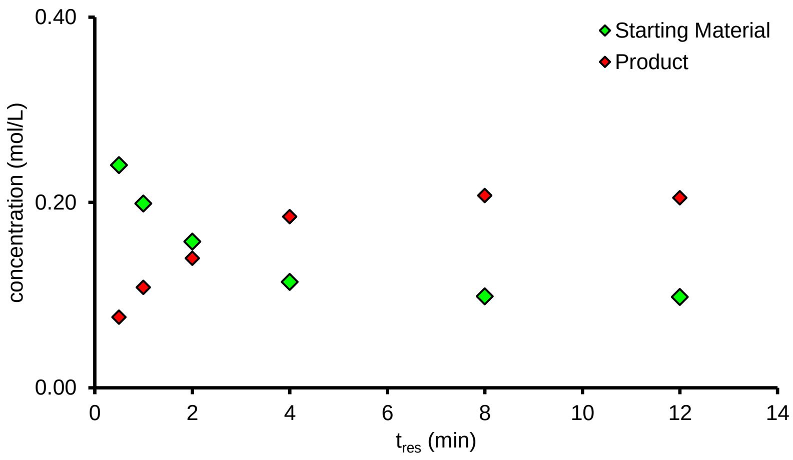

Figure S35. Time course data obtained from experiment series 2.

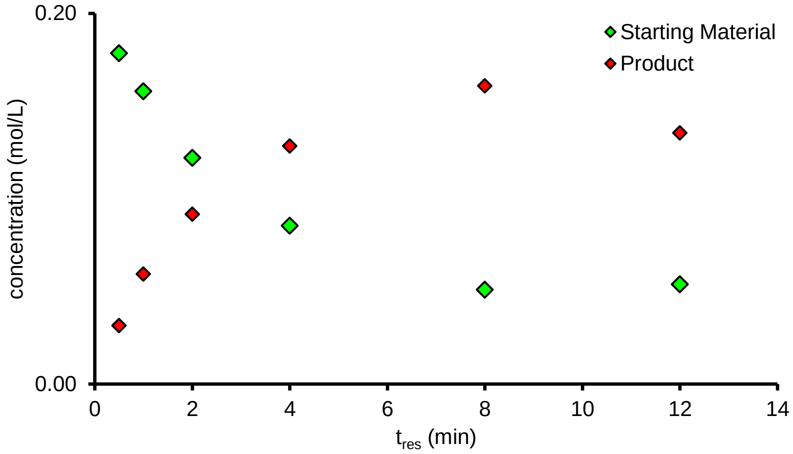

Figure S36 Time course data obtained from experiment series 3.

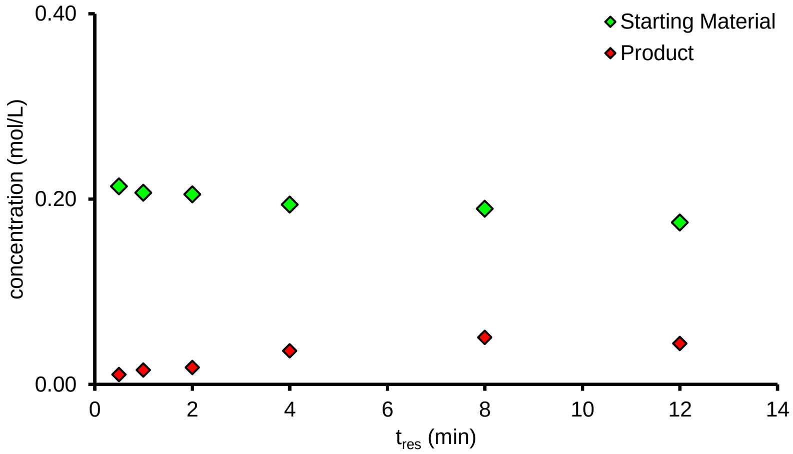

Figure S37. Time course data obtained from experiment series 4.

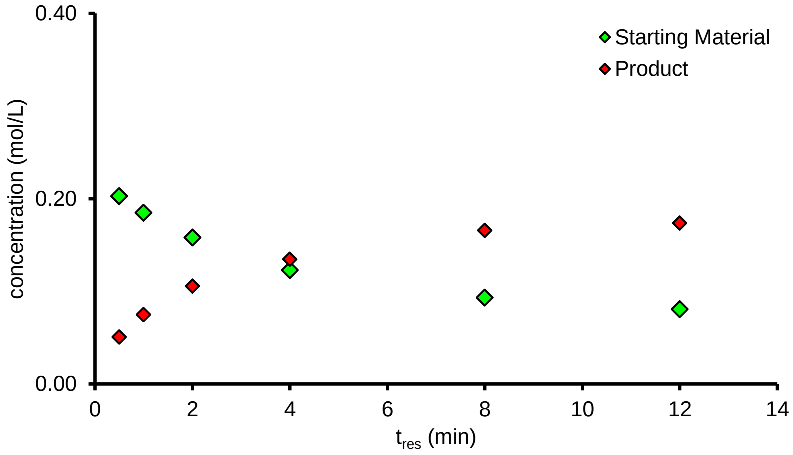

Figure S38 Time course data obtained from experiment series 5.

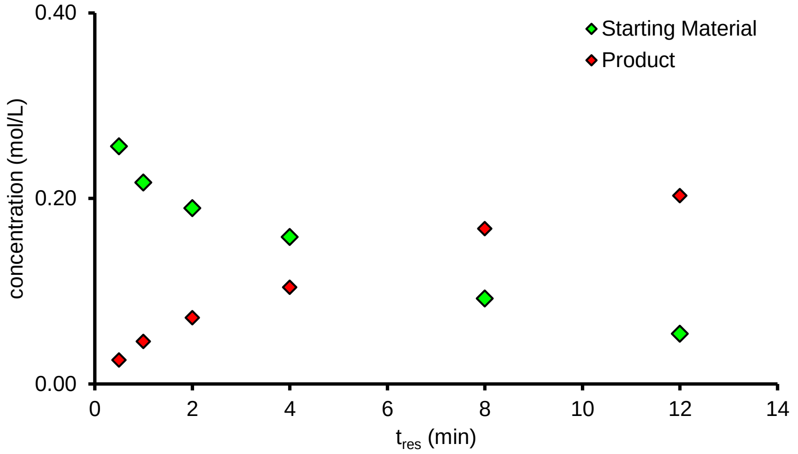

Figure S39. Time course data obtained from experiment series 6.

Table S9. Results from the kinetic study performed using information from the DoE to decide boundary conditions. The adjusted variables were the ratio of Amine starting material to ArBr starting material, concentration of ArBr starting material, the temperature of the reactor, the equivalents of base and the mol % of catalyst. Each set of conditions was measured at 6 different residence times.

#	Amine **2** loading	c_SM	RT	Temp	DBU Loading	Cat. loading	Yield	[Product **3**]	SM
	(equiv)	(mol/L)	(min)	(°C)	(equiv)	(mol%)	(%)	(mol/L)	(mol/L)
1	1.30	0.323	0.5	140	1.63	5.11	32.1	0.10	0.203
	1.30	0.323	1	140	1.63	5.11	46.9	0.15	0.150
	1.30	0.323	2	140	1.63	5.11	64.2	0.21	0.094
	1.30	0.323	4	140	1.63	5.11	88.3	0.29	0.021
	1.30	0.323	8	140	1.63	5.11	93.7	0.30	0.007
	1.30	0.323	12	140	1.63	5.11	93.8	0.30	0.008
2	1.30	0.323	0.5	140	0.82	5.11	23.6	0.08	0.240
	1.30	0.323	1	140	0.82	5.11	33.6	0.11	0.199
	1.30	0.323	2	140	0.82	5.11	43.4	0.14	0.158
	1.30	0.323	4	140	0.82	5.11	57.3	0.18	0.115
	1.30	0.323	8	140	0.82	5.11	64.4	0.21	0.099
	1.30	0.323	12	140	0.82	5.11	63.7	0.21	0.098
3	1.09	0.231	0.5	125	1.63	5.43	13.7	0.03	0.179
	1.09	0.231	1	125	1.63	5.43	25.8	0.06	0.158
	1.09	0.231	2	125	1.63	5.43	39.8	0.09	0.122
	1.09	0.231	4	125	1.63	5.43	55.8	0.13	0.086
	1.09	0.231	8	125	1.63	5.43	69.9	0.16	0.051
	1.09	0.231	12	125	1.63	5.43	58.8	0.14	0.054
4	1.09	0.231	0.5	130	1.63	2.72	4.6	0.01	0.214
	1.09	0.231	1	130	1.63	2.72	6.7	0.02	0.207
	1.09	0.231	2	130	1.63	2.72	7.9	0.02	0.205
	1.09	0.231	4	130	1.63	2.72	15.7	0.04	0.194
	1.09	0.231	8	130	1.63	2.72	22.0	0.05	0.190
	1.09	0.231	12	130	1.63	2.72	19.1	0.04	0.175
5	0.87	0.268	0.5	135	1.09	4.89	18.9	0.05	0.203
	0.87	0.268	1	135	1.09	4.89	28.0	0.07	0.185
	0.87	0.268	2	135	1.09	4.89	39.5	0.11	0.158
	0.87	0.268	4	135	1.09	4.89	50.3	0.13	0.123
	0.87	0.268	8	135	1.09	4.89	61.9	0.17	0.093
	0.87	0.268	12	135	1.09	4.89	64.8	0.17	0.081
6	1.20	0.268	0.5	130	1.63	3.26	9.7	0.03	0.256
	1.20	0.268	1	130	1.63	3.26	17.2	0.05	0.217
	1.20	0.268	2	130	1.63	3.26	26.8	0.07	0.190
	1.20	0.268	4	130	1.63	3.26	39.0	0.10	0.159
	1.20	0.268	8	130	1.63	3.26	62.7	0.17	0.092
	1.20	0.268	12	130	1.63	3.26	76.0	0.20	0.054

Note: experiment 4 was not included in model parameterization, since its low catalyst loading resulted in far poorer performance than the other five experiments. This observation could not be easily accounted for in a simple reaction model.

9.1. Reaction Catalytic Cycle and Kinetic Model

Based on the data gathered in kinetic experiments (**Table S9**), a kinetic model was proposed and parameterized. **At this point, it should be stated that this does not intend to act as a mechanistically accurate model, but simply to describe the observed reaction outcomes and develop a predictive model for the outcome of other reaction conditions.**

The catalytic cycle used in the kinetic model is shown in **Figure S40**, based on literature reports.[2][3] In this model cycle, the rate limiting step is the oxidative addition to the Pd catalyst (k_{addn}), with both activation energy and pre-exponential factor fitted. The rates for the amine association, bromide elimination and reductive elimination are fixed at a high value ($k = 10^9$).

Catalyst activation and ligation equilibria[2] were examined, but were found to have no impact on the model fitting. This is likely due to the fact that the catalyst and ligand were premixed in one feed solution for these experiments. As a result, these were removed from the kinetic model, simply treating the catalyst supplied to the reactor as "PdL".

In order to fit the experimental observations more closely, one single pathway has been added to account for catalyst deactivation and all impurity formation. This was modelled using a temperature-independent rate constant, k_{dec}.

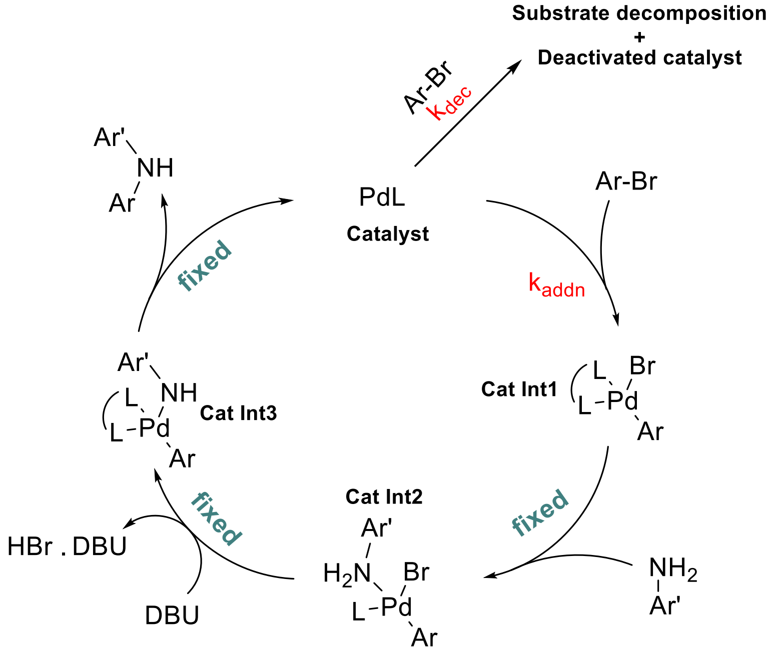

Figure S40. Scheme of the model catalytic cycle with fitted parameters shown in red.

The considered reaction steps are summarized below:

Oxidative addition: $\frac{d[Cat\ Int1]}{dt} = k_{addn}[Catalyst][ArBr] = Ae^{\frac{-Ea}{RT}}[Catalyst][ArBr]$

Amine coordination: $\frac{d[Cat\ Int2]}{dt} = 10^9[CatInt1][Amine]$

Halide abstraction: $\frac{d[Cat\ Int3]}{dt} = 10^9[CatInt2][DBU]$

Reductive elimination: $\frac{d[Product]}{dt} = 10^9[CatInt3]$

Substrate decomposition: $\frac{d[Decomposition]}{dt} = k_{dec}[Catalyst][ArBr]$

The three parameters (shown in red) were fitted simultaneously, using the dataset shown above (Table S9), excluding experiment 4 due to the low catalyst loading giving non-representative results. The following parameter values were found:

Table S10. Values fitted for the kinetic model, based on the catalytic cycle shown above.

Parameter	Value	Coefficient of Variation
Ea	24.05 kJ/mol	29.5%
A	771.2	207%
k_{dec1}	0.03570	10.9%

It should be noted that Ea and A have a large coefficient of variation because the two parameters are highly correlated, which allows a significant extent of flexibility between the values. Fitting a single temperature-independent rate constant (k_{addn}) provides a far lower coefficient of variation (~10%), but does not account for any temperature dependence in the reaction data.
As stated above, these values are not intended to be mechanistically accurate, but to simply provide a useful predictive model.

The kinetic model was then validated, using results from the self-optimization and DoE data sets.

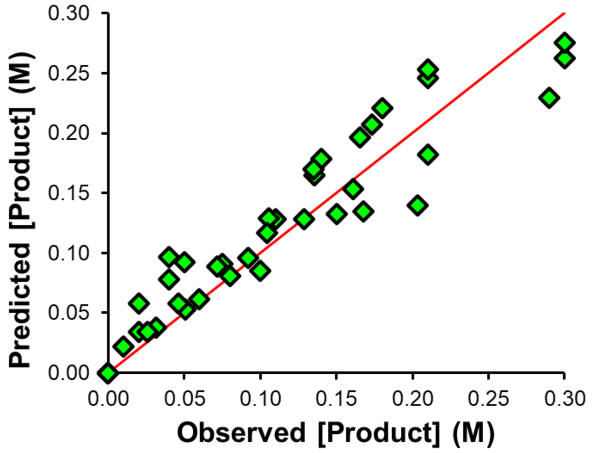

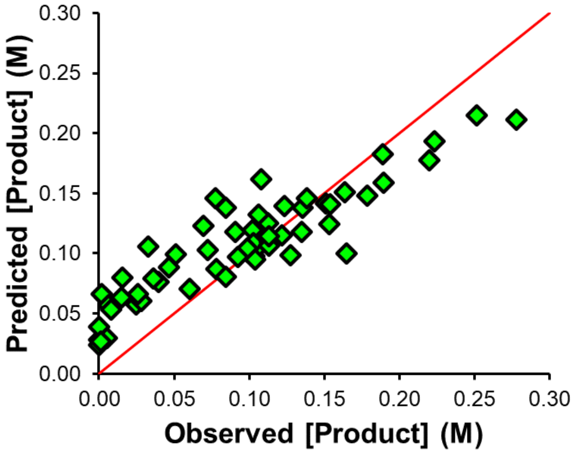

Figure S41. Parity plots showing the product concentration predicted by the kinetic model vs product concentration observed experimentally. ***Left:*** *Parity plot for model fitting data, R^2 = 0.8884.* ***Right:*** *parity plot for self-optimization results, R^2 = 0.7245.*

Table S11. Results of kinetic model predictions for self-optimization experiment results, compared with the experimentally-measured values, showing the calculated residual for each result. RMSE = 34.9 mM.

Experiment number	Amine **2** Loading	c_SM	RT	Temp	DBU Loading	Cat. loading	Measured [Product **3**]	Predicted [Product **3**]	Residual [Product **3**]
	(equiv)	(mol/L)	(min)	(°C)	(equiv)	(mol%)	(mol/L)	(mol/L)	(mol/L)
1	1.46	0.234	9.0	100.4	1.18	1.50	0.000	0.028	0.0283
2	1.25	0.279	2.2	105.3	1.63	3.45	0.025	0.058	0.0333
3	1.16	0.222	6.0	107.9	1.10	3.26	0.028	0.061	0.0324
4	1.14	0.320	3.8	112.6	1.31	5.21	0.151	0.141	-0.0094
5	1.34	0.247	3.0	115.4	2.13	1.13	0.000	0.024	0.0239
6	1.31	0.259	7.3	118.3	1.51	2.18	0.004	0.064	0.0598
7	1.10	0.299	4.3	120.0	1.90	1.95	0.015	0.063	0.0484
8	1.20	0.305	9.4	124.4	1.59	4.13	0.190	0.159	-0.0308
9	1.24	0.268	4.8	128.4	1.40	4.67	0.135	0.138	0.0026
10	1.39	0.344	6.3	130.7	1.70	0.59	0.005	0.030	0.0243
11	1.43	0.209	8.5	136.5	1.83	2.58	0.015	0.080	0.0643
12	1.41	0.332	7.4	137.0	1.99	4.30	0.278	0.211	-0.0666
13	1.30	0.333	7.8	105.5	1.38	2.16	0.002	0.066	0.0641
14	1.22	0.200	7.4	105.4	1.36	2.30	0.000	0.039	0.0384
15	1.20	0.230	6.3	120.2	1.84	4.70	0.107	0.111	0.0039
16	1.07	0.228	9.9	132.2	1.18	2.79	0.046	0.089	0.0424
17	1.33	0.350	9.7	104.5	1.07	4.00	0.112	0.125	0.0124
18	1.26	0.223	10.0	140.0	1.07	4.76	0.179	0.148	-0.0308
19	1.13	0.257	8.4	140.0	1.07	3.84	0.139	0.146	0.0070
20	1.29	0.244	2.0	127.0	1.10	3.55	0.026	0.066	0.0401
21	1.09	0.296	5.5	125.6	1.17	3.41	0.102	0.120	0.0175
22	1.21	0.341	3.9	129.6	1.11	2.95	0.069	0.123	0.0536
23	1.24	0.230	2.8	138.9	1.39	4.20	0.103	0.098	-0.0056
24	1.17	0.305	3.9	140.0	1.16	0.57	0.001	0.027	0.0252
25	1.48	0.281	4.0	137.7	1.23	2.85	0.033	0.105	0.0729
26	1.50	0.272	5.4	126.9	1.29	5.06	0.164	0.151	-0.0125
27	1.50	0.224	2.9	131.9	1.40	4.19	0.078	0.087	0.0085
28	1.35	0.205	2.0	136.3	1.07	5.30	0.084	0.081	-0.0036
29	1.09	0.217	3.8	140.0	1.59	4.03	0.165	0.100	-0.0644
30	1.14	0.200	5.7	140.0	1.07	4.15	0.104	0.103	-0.0013
31	1.07	0.212	5.4	137.4	1.10	2.74	0.040	0.076	0.0365
32	1.30	0.250	4.9	130.0	1.70	4.10	0.135	0.118	-0.0168
33	1.33	0.215	3.8	139.3	1.23	5.13	0.122	0.115	-0.0068
34	1.30	0.207	9.8	130.0	1.07	4.24	0.102	0.109	0.0062
35	1.07	0.226	4.4	135.9	1.51	3.83	0.099	0.101	0.0029

36	1.08	0.238	7.9	140.0	1.49	2.68	0.051	0.099	0.0483
37	1.13	0.215	4.6	134.5	1.64	4.58	0.114	0.109	-0.0045
38	1.11	0.211	4.9	131.5	1.45	3.25	0.036	0.079	0.0429
39	1.07	0.221	3.9	139.2	1.07	3.94	0.127	0.099	-0.0284
40	1.07	0.240	5.9	139.6	1.07	4.41	0.084	0.138	0.0535
41	1.08	0.269	6.3	112.2	1.07	4.10	0.072	0.103	0.0311
42	1.07	0.210	7.7	138.0	1.88	4.07	0.091	0.118	0.0271
43	1.09	0.350	8.9	136.4	1.12	2.98	0.108	0.162	0.0540
44	1.18	0.262	6.2	137.3	1.92	5.35	0.220	0.177	-0.0428
45	1.07	0.200	8.5	137.4	1.15	5.34	0.106	0.132	0.0262
46	1.18	0.214	7.2	139.1	1.47	4.34	0.153	0.125	-0.0286
47	1.12	0.223	2.1	137.5	1.49	5.27	0.104	0.095	-0.0094
48	1.17	0.229	4.6	129.3	1.11	2.14	0.006	0.057	0.0503
49	1.12	0.226	6.5	140.0	1.38	3.32	0.113	0.107	-0.0067
50	1.14	0.336	2.1	136.7	1.32	4.01	0.154	0.141	-0.0134
51	1.11	0.350	2.3	121.9	1.09	4.66	0.123	0.139	0.0159
52	1.18	0.208	2.0	140.0	1.18	4.14	0.060	0.071	0.0104
53	1.50	0.287	4.4	140.0	1.15	5.21	0.189	0.183	-0.0066
54	1.15	0.285	3.3	139.0	1.62	3.18	0.113	0.114	0.0008
55	1.22	0.243	2.3	130.6	1.07	2.45	0.008	0.053	0.0447
56	1.29	0.206	5.5	122.5	1.50	5.31	0.098	0.104	0.0056
57	1.23	0.342	4.0	136.0	1.48	4.47	0.223	0.193	-0.0299
58	1.13	0.200	5.9	132.5	2.01	4.11	0.092	0.097	0.0044
59	1.23	0.339	4.5	134.2	1.07	5.33	0.251	0.215	-0.0368
60	1.12	0.279	4.4	126.8	1.11	5.04	0.078	0.146	0.0680

Table S12. Results of kinetic model predictions for DoE results, compared with the experimentally-measured values, showing the calculated residual for each result. RMSE = 25.0 mM.

Experiment number	Amine **2** Loading	c_SM	RT	Temp	DBU Loading	Cat. loading	Measured [Product **3**]	Predicted [Product **3**]	Residual [Product **3**]
	(equiv)	(mol/L)	(min)	(°C)	(equiv)	(mol%)	(mol/L)	(mol/L)	(mol/L)
1	1.18	0.35	6.5	135	1.34	4.82	0.233	0.234	0.0012
2	1.07	0.35	6.5	130	1.07	4.28	0.191	0.198	0.0072
3	1.07	0.35	6.5	130	1.07	5.35	0.196	0.234	0.0389
4	1.28	0.35	6.5	130	1.07	5.35	0.204	0.234	0.0306
5	1.07	0.35	6.5	130	1.61	4.28	0.226	0.202	-0.0241
6	1.07	0.35	6.5	130	1.61	5.35	0.242	0.239	-0.0036
7	1.28	0.35	6.5	130	1.61	4.28	0.238	0.202	-0.0360
8	1.18	0.35	6.5	130	1.34	4.82	0.218	0.220	0.0024
9	1.18	0.35	6.5	135	1.34	4.28	0.225	0.215	-0.0097
10	1.18	0.35	6.5	135	1.34	5.35	0.249	0.251	0.0021
11	1.07	0.35	6.5	135	1.34	4.82	0.229	0.234	0.0053
12	1.28	0.35	6.5	135	1.34	4.82	0.255	0.234	-0.0213
13	1.18	0.35	6.5	135	1.34	4.82	0.252	0.234	-0.0174
14	1.18	0.35	6.5	135	1.34	4.82	0.244	0.234	-0.0100
15	1.18	0.35	6.5	135	1.07	4.82	0.214	0.231	0.0167
16	1.18	0.35	6.5	135	1.61	4.82	0.270	0.231	-0.0391
17	1.07	0.35	6.5	140	1.07	4.28	0.223	0.225	0.0024
18	1.28	0.35	6.5	140	1.07	4.28	0.224	0.225	0.0012
19	1.28	0.35	6.5	140	1.07	5.35	0.234	0.260	0.0264
20	1.07	0.35	6.5	140	1.61	5.35	0.265	0.265	-0.0001
21	1.28	0.35	6.5	140	1.61	4.28	0.288	0.230	-0.0573
22	1.28	0.35	6.5	140	1.61	5.35	0.299	0.265	-0.0340
23	1.18	0.35	6.5	140	1.34	4.82	0.266	0.248	-0.0181
24	1.18	0.35	6.5	135	1.34	4.82	0.263	0.234	-0.0286
25	1.18	0.35	6.5	135	1.34	4.82	0.246	0.234	-0.0114
26	1.18	0.35	6.5	135	1.34	4.82	0.249	0.221	-0.0277
27	1.07	0.35	6.5	140	1.07	5.35	0.227	0.246	0.0190
28	1.07	0.35	6.5	140	1.61	4.28	0.266	0.218	-0.0477
29	1.18	0.35	6.5	135	1.34	4.82	0.247	0.221	-0.0251
30	1.28	0.35	6.5	130	1.07	4.28	0.199	0.188	-0.0117
31	1.28	0.35	6.5	130	1.61	5.35	0.266	0.226	-0.0402

10. Continuous Flow Validation & Scale-Up Experiments

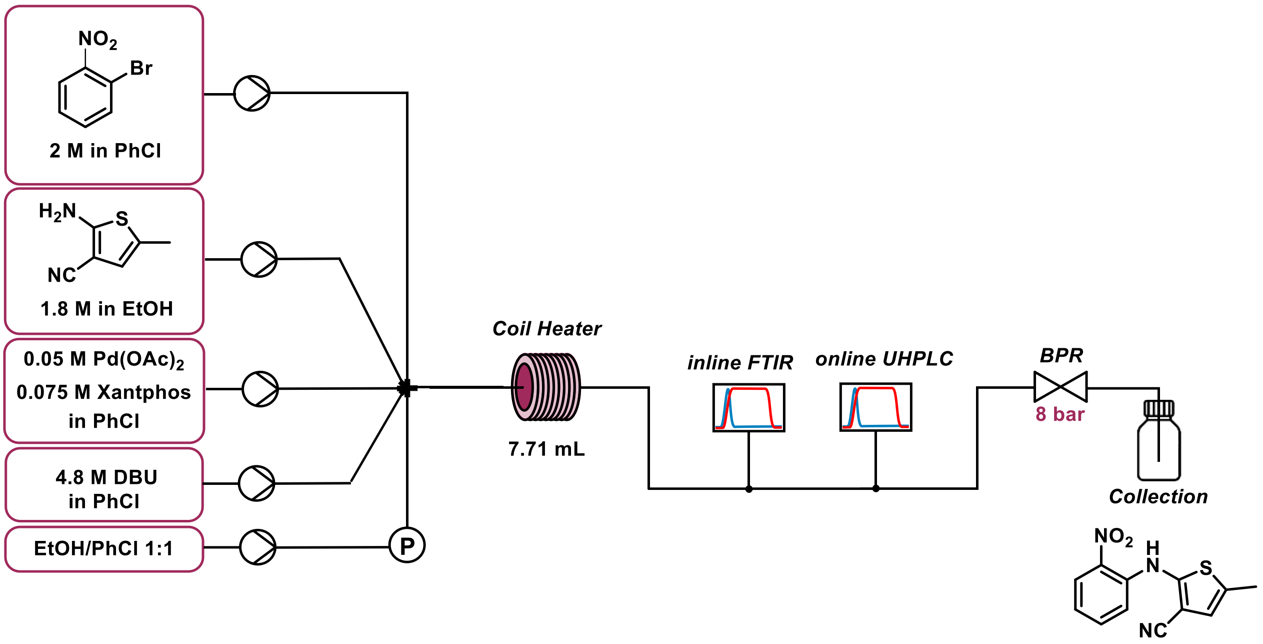

Figure S42. Detailed flow Setup for the continuous validation & scale-up runs. "P" denotes a pressure sensor.

The scaled-up validation reaction was carried out in a Uniqsis coil reactor using 1/8" PFA tubing according to the flow scheme shown in **Figure S42**. The feeds were delivered continuously using five Knauer AZURA P 4.1S HPLC pumps. The solvent pump was connected to a pressure sensor (Keller, PAA 35XHTC). The streams were combined using a 7-way mixer (IDEX P-151, 83 µL i.V.) with one of the ports blocked off with a PEEK stopper. After passing the heated coil reactor (7.71 mL), the process stream was transported to the FTIR using a spacer of 1/8"PFA tubing and 0.1 mm PTFE tubing. After the FTIR, the process stream was transported into the injection valve of the UHPLC using 0.1 mm PTFE tubing. The stream was continuously sampled until the conclusion of the experiment. After passing the UHPLC the process stream was transported to a membrane-based BPR (Zaiput, BPR-10, set to 8 bars) in 1/16" PFA tubing and collected after passing through the BPR.

The continuous runs were performed based on the result of the self-optimization, DoE model and kinetic model using the parameters shown in **Table S13**. Results are shown in **Figure S43**, **Figure S44** and **Figure S45**.

Table S13. Parameters for continuous flow runs.

Conditions	Based on	Amine **2** Loading (equiv)	t_{res} (min)	c_SM (mol/L)	T (°C)	DBU Loading (equiv)	Catalyst loading (mol %)	Average measured yield (%)	Predicted yield (%)	Discrepancy (%)
1	Self-optimization	1.18	6.2	0.24	137	1.92	5.35	77.4	91.1	13.7
2	DoE	1.28	6.5	0.33	140	1.61	4.92	83.5	90.2	6.7
3	Kinetic model	1.28	4.2	0.33	140	1.61	5.35	70.1	74.2	4.1

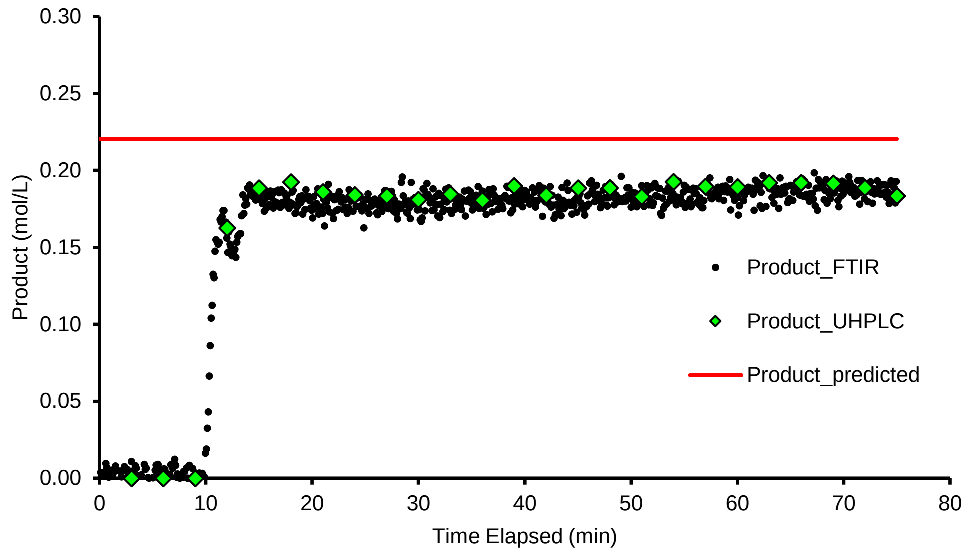

Figure S43. Product concentration as determined by FTIR and UHPLC over time using conditions 1, inputs chosen based on self-optimization results. Baseline correction has been applied to FTIR data.

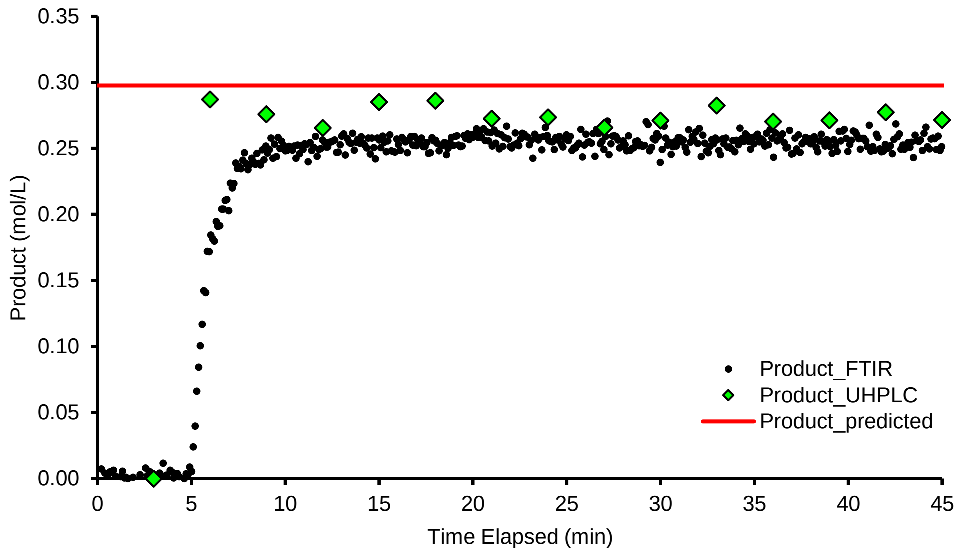

Figure S44. Product concentration as determined by FTIR and UHPLC over time using conditions 2, inputs chosen based on DoE results. Baseline correction has been applied to FTIR data.

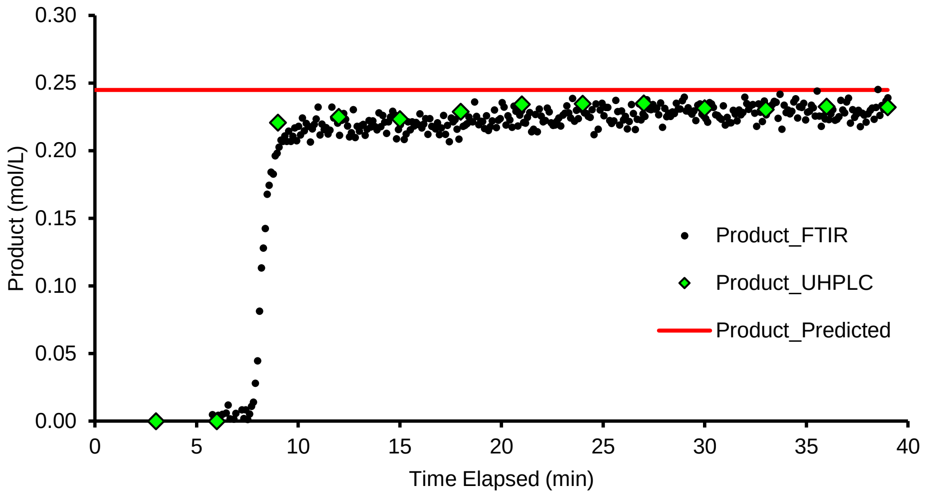

Figure S45. Product concentration as determined by FTIR and UHPLC over time using conditions 3, inputs chosen based on kinetic model. Baseline correction has been applied to FTIR data.

11. Batch Synthesis of Product

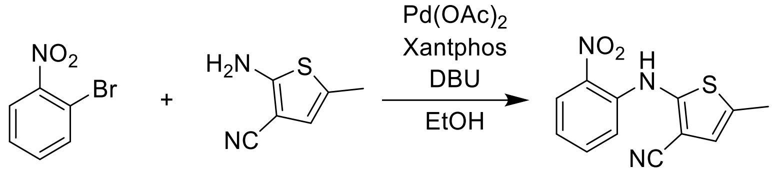

1 2 3

For use in UHPLC calibration, Product **3** was synthesized using a modified literature protocol.[4]

A 16 mL microwave vial fitted with a magnetic stirring bar was charged with **1** (800 mg, 3.96 mmol) and **2** (548 mg, 3.97 mmol), $Pd(OAc)_2$ (44.7 mg, 0.20 mmol) and Xantphos (126 mg, 0.22 mmol). The solids were suspended in EtOH (12 mL, 99.7%) and DBU (1.2 mL, 0.049 mmol) was added. The microwave vial was heated to 130 °C for 10 minutes using the Anton Paar Monowave 300 microwave system. Reaction progress was monitored using HPLC. After allowing the reaction mixture to cool to room temperature H_2O (10 mL) was added. The aqueous phase was extracted using DCM (3 x 10 mL) and the combined organic phases were dried over Na_2SO_4. The dried organic phases were evaporated to afford the crude coupling product (**3**). The product was purified using flash column chromatography (EtOAc:Cyclohexane 3:1 + 5% Et_3N) to afford **3** (361 mg, 1.39 mmol, 34% yield, 86% purity by HPLC).

12. References

[1] A. M. Schweidtmann, A. D. Clayton, N. Holmes, E. Bradford, R. A. Bourne, A. A. Lapkin, *Chem. Eng. J.* **2018**, *352*, 277–282.

[2] S. Shekhar, P. Ryberg, J. F. Hartwig, J. S. Mathew, D. G. Blackmond, E. R. Strieter, S. L. Buchwald, *J. Am. Chem. Soc.* **2006**, *128*, 3584–3591.

[3] R. Dorel, C. P. Grugel, A. M. Haydl, *Angew. Chem. Int. Ed.* **2019**, *58*, 17118–17129.

[4] D. Obermayer, D. Znidar, G. Glotz, A. Stadler, D. Dallinger, C. Oliver Kappe, *J. Org. Chem.* **2016**, *81*, 11788–11801.
